# Supplementary material for: Guidance for good practice in the application of machine learning in development of toxicological quantitative structure-activity relationships (QSARs)
Source: PLoS One. 2023 May 10;18(5):e0282924. doi: 10.1371/journal.pone.0282924 (PMC10171609; doi:10.1371/journal.pone.0282924)

**Supplementary Material 2.** Hyperparameter optimisation (manual)

Optimisation curves displayed within figures below outline influence upon the model performance metrics R^2^_train_ and R^2^_CV_ (k = 10), arising as a consequence of the incremental variation in value of a given hyperparameter over its defined range (with all others simultaneously held constant at their default quantities).

The following legend is relatable to all:


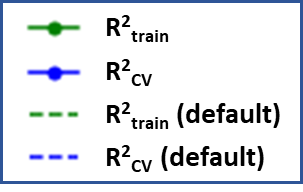


Tables list both default and identified optimal values relating to each examined parameter. Upper and lower boundaries of ranges assessed during the search process are provided, as are step sizes separating those quantities eligible for consideration. R^2^_train_ and R^2^_CV_ correspond to performance of model trained through use of the optimum hyperparameter value.

**Random forest**

| **Parameter** | **Default** | **Start** | **End** | **Step size** | **Optimal** | **R^2^ _train_** | **R^2^_CV_** |
| --- | --- | --- | --- | --- | --- | --- | --- |
| max_depth | Automatic* | 1 | 50 | 1 | 15 | 0.962 | 0.751 |
| min_samples_split | 2 | 2 | 20 | 1 | 3 | 0.963 | 0.751 |
| min_samples_leaf | 1 | 1 | 100 | 10 | 1 | 0.964 | 0.750 |
| max_leaf_nodes | Automatic* | 2 | 202 | 10 | Automatic | 0.952 | 0.747 |
| n_estimators | 100 | 50 | 500 | 10 | 490 | 0.966 | 0.753 |
| max_samples | Automatic* | 0.1 | 0.99 | 0.1 | 0.99 | 0.964 | 0.752 |

* Value of parameter defined by algorithm should the term “None” be entered (please refer to official scikit-learn documentation, linked within Section 2.5 of manuscript).


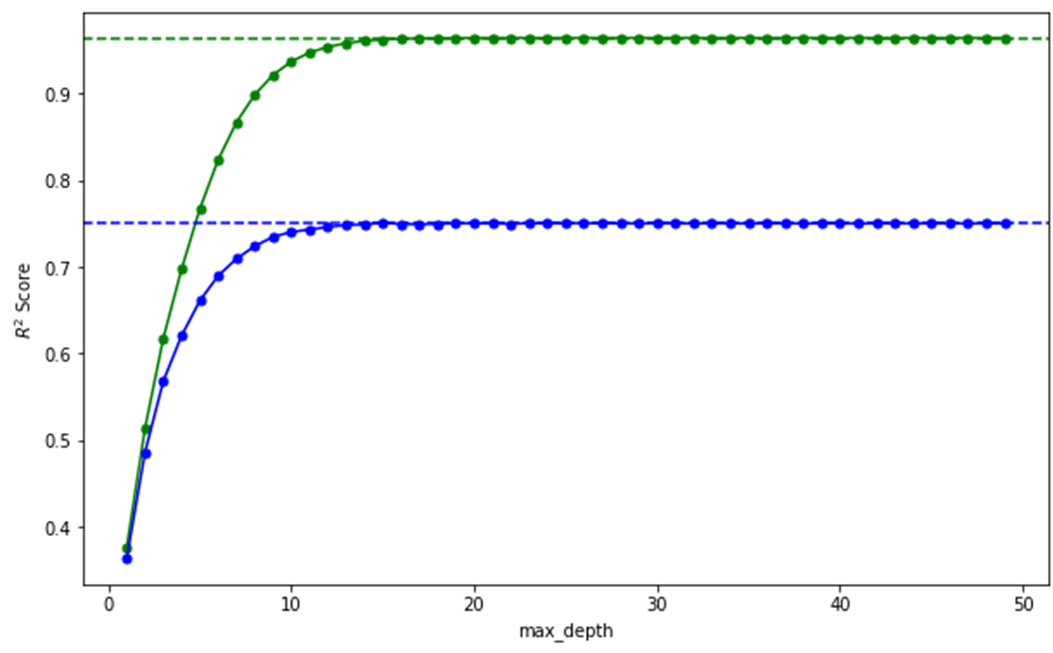


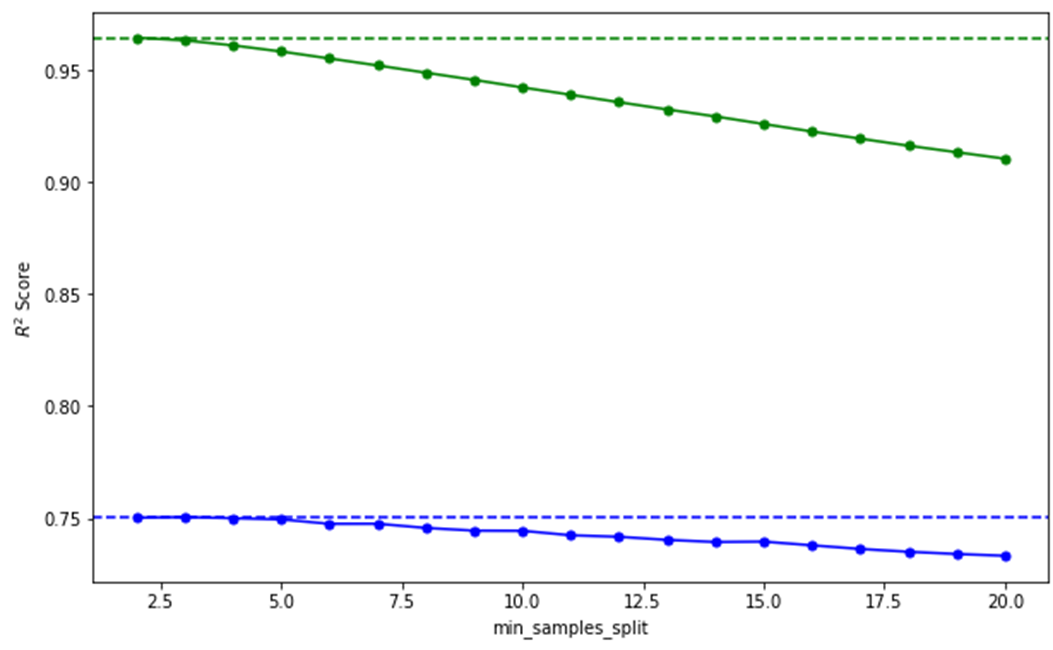


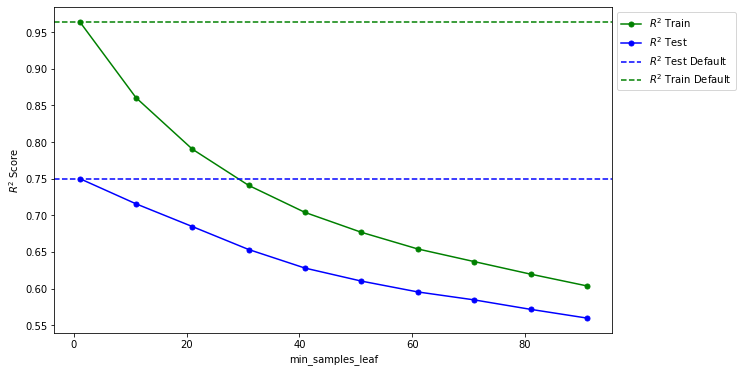


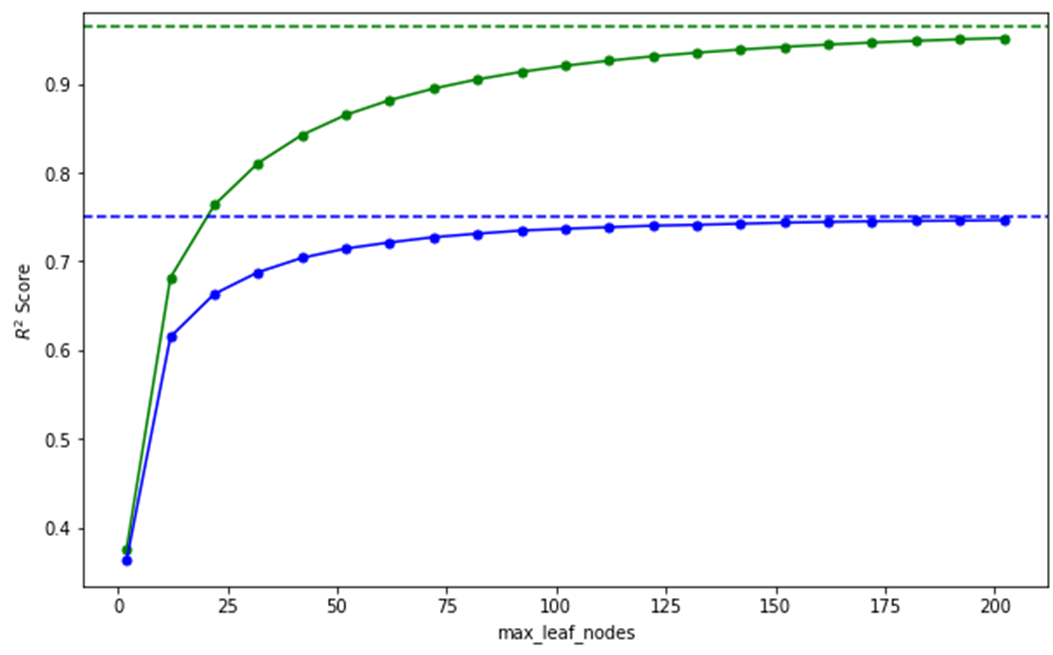


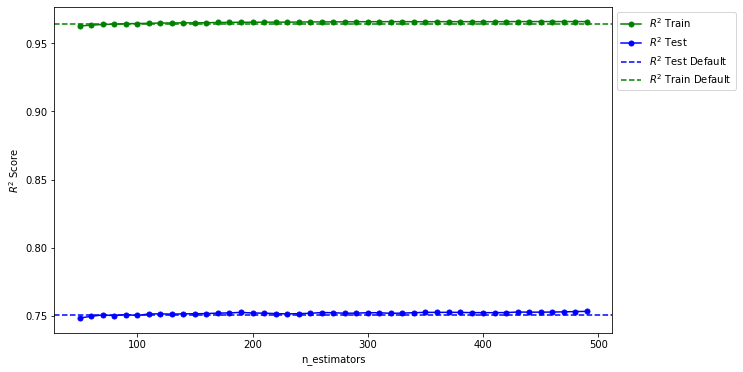


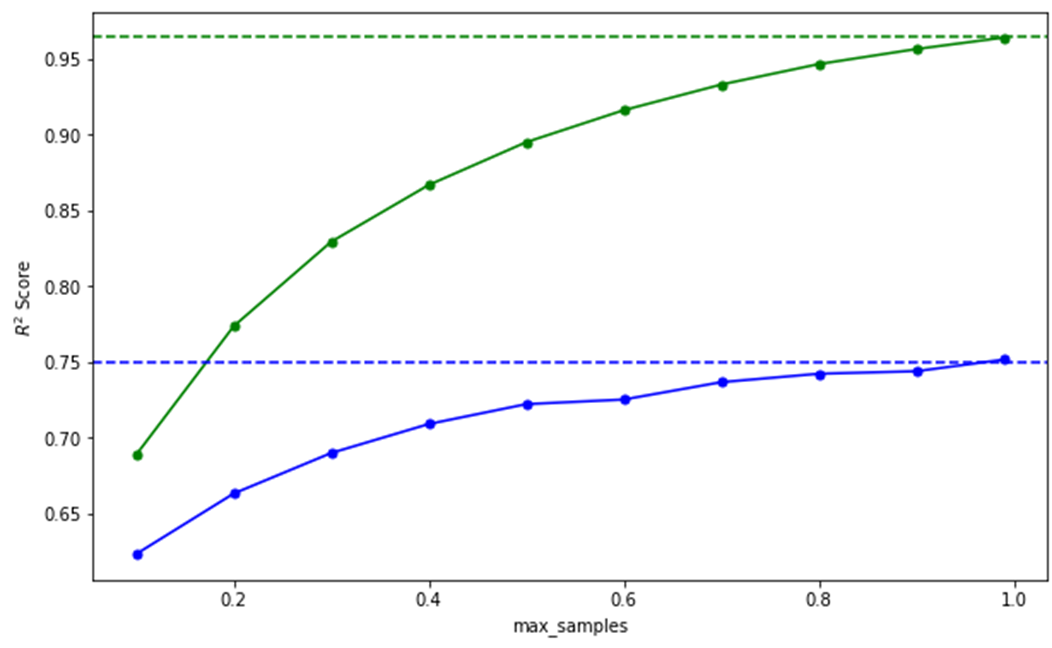


**Support vector machine**

| **Parameter** | **Default** | **Start** | **End** | **Step size** | **Optimal** | **R^2^ _train_** | **R^2^_CV_** |
| --- | --- | --- | --- | --- | --- | --- | --- |
| gamma | scale* | 0.01 | 0.0001 | 3.81E-04 | 0.002 | 0.883 | 0.751 |
| C | 1 | 0.5 | 50 | Irregular† | 5 | 0.981 | 0.780 |
| epsilon | 0.1 | 0.001 | 1 | 0.02 | 0.042 | 0.902 | 0.746 |

* Value of parameter defined automatically by algorithm (please refer to official scikit-learn documentation, linked within Section 2.5 of manuscript).

† Values of C tested = 0.5, 0.6, 0.7, 0.8, 0.9, 1, 2, 3, 4, 5, 6, 7, 8, 9, 10, 20, 30, 40, 50


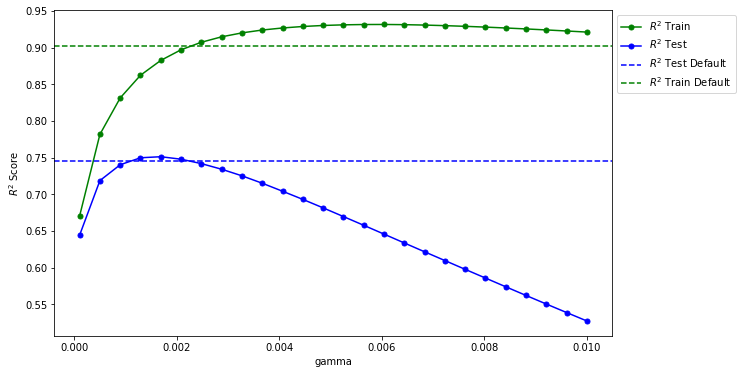


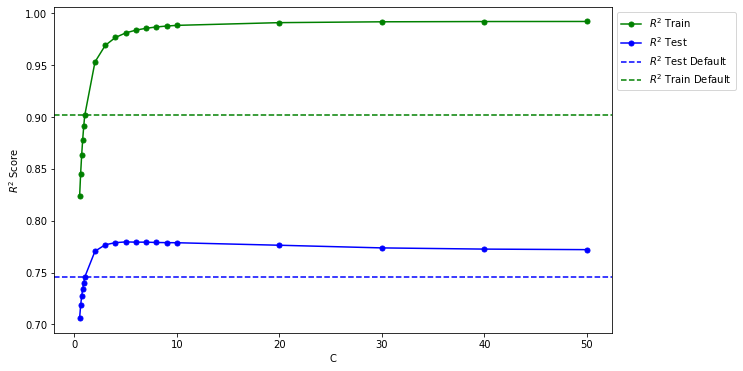


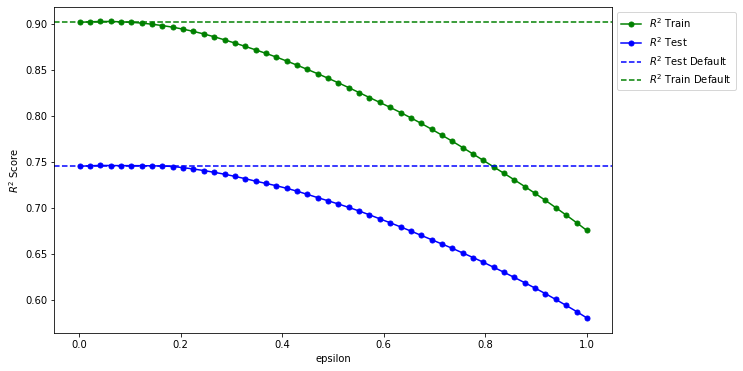


***k*-Nearest neighbours**

| **Parameter** | **Default** | **Start** | **End** | **Step size** | **Optimal** | **R^2^ _train_** | **R^2^_CV_** |
| --- | --- | --- | --- | --- | --- | --- | --- |
| n_neighbors | 5 | 1 | 20 | 1 | 6 | 0.767 | 0.662 |
| p | 2 | 1 | 5 | 1 | 1 | 0.803 | 0.694 |


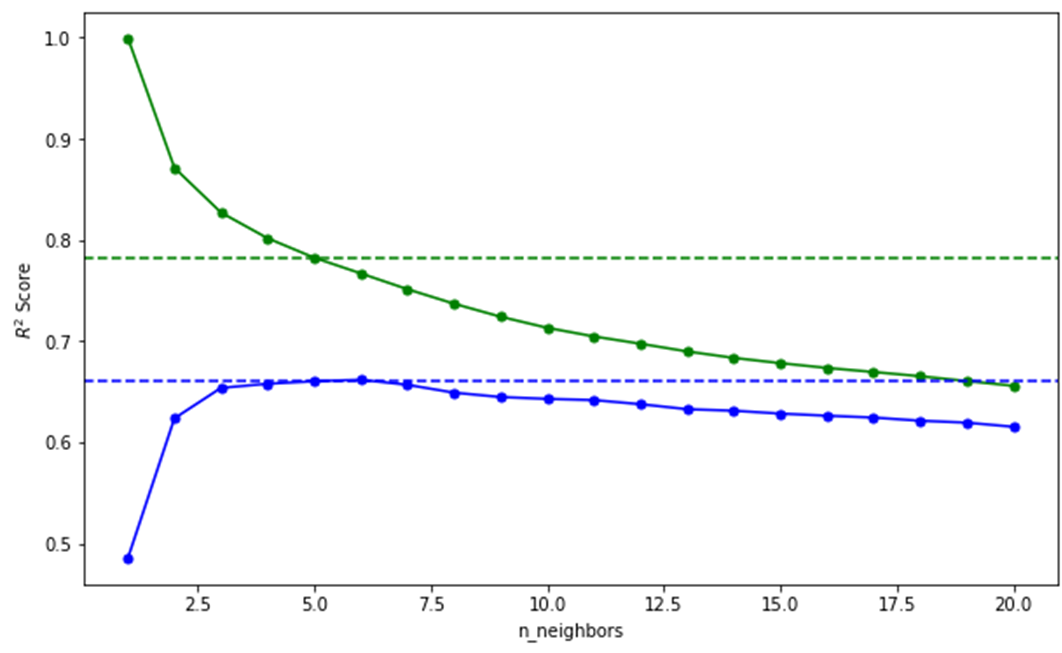


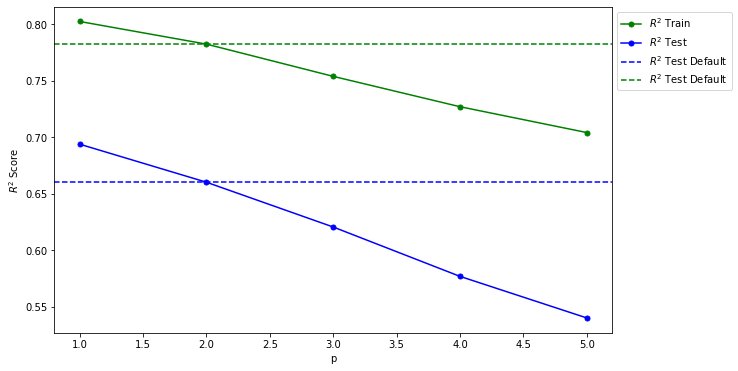


**Extreme gradient boosting**

| **Parameter** | | **Default** | | **Start** | | | **End** | | **Step size** | | **Optimal** | | **R^2^ _train_** | **R^2^_CV_** | |
| --- | --- | --- | --- | --- | --- | --- | --- | --- | --- | --- | --- | --- | --- | --- | --- |
| eta | | 0.3 | | 0.005 | | | 0.5 | | 0.0165 | | 0.107 | | 0.992 | 0.798 | |
| min_child_weight | | 1 | | 1 | | | 20 | | 1 | | 7 | | 0.999 | 0.783 | |
| max_depth | | 6 | | 1 | | | 50 | | 1 | | 4 | | 0.990 | 0.780 | |
| gamma | | 0 | | 0 | | | 3 | | 0.1 | | 0.103 | | 0.990 | 0.780 | |
| n_estimators | | 100 | | 50 | | | 500 | | 10 | | 250 | | 1.000 | 0.779 | |
| subsample | | 1 | | 0.1 | | | 1 | | 0.1 | | 1 | | 1.000 | 0.778 | |
| colsample_bytree | | 1 | | 0.1 | | | 1 | | 0.1 | | 0.6 | | 1.000 | 0.780 | |
| max_delta_step | | 0 | | 0 | | | 10 | | 1 | | 0 | | 1.000 | 0.778 | |
| lambda | | 1 | | 0 | | | 1 | | 0.1 | | 0.778 | | 1.000 | 0.778 | |
| alpha | | 0 | | 0 | | | 10 | | 1 | | 3 | | 0.992 | 0.784 | |
|  |  | |  | |  |  | |  | |  | |  | | |  |


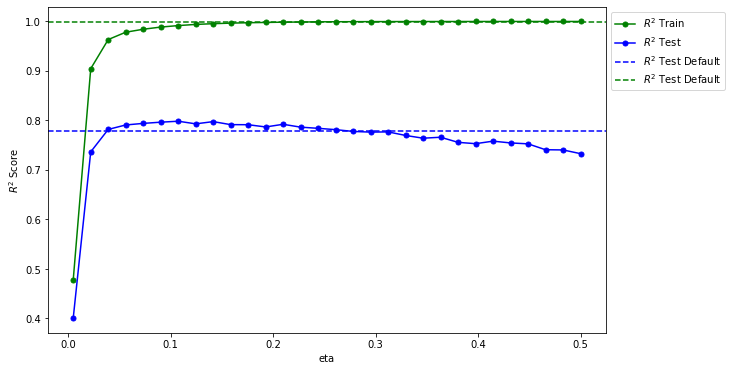


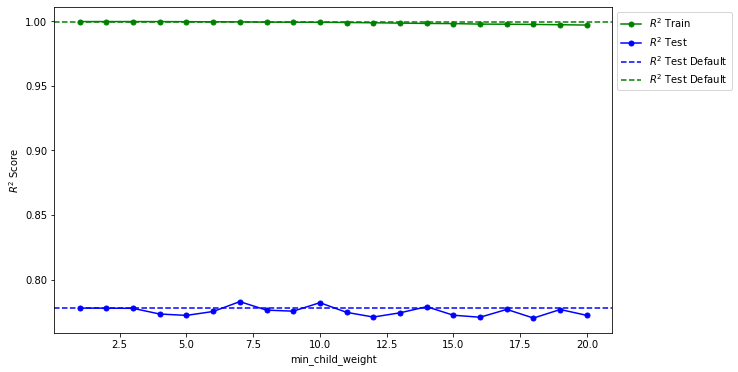


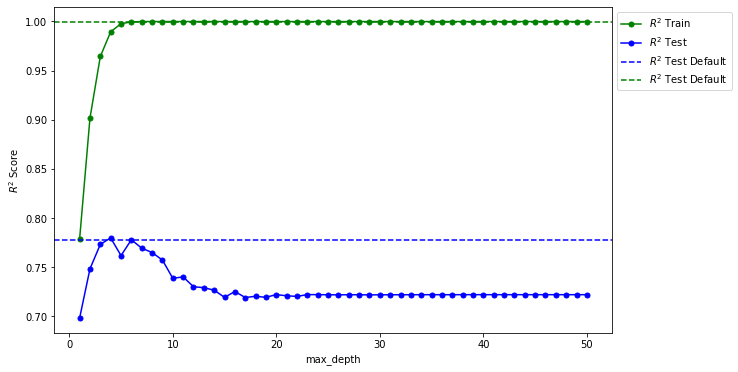


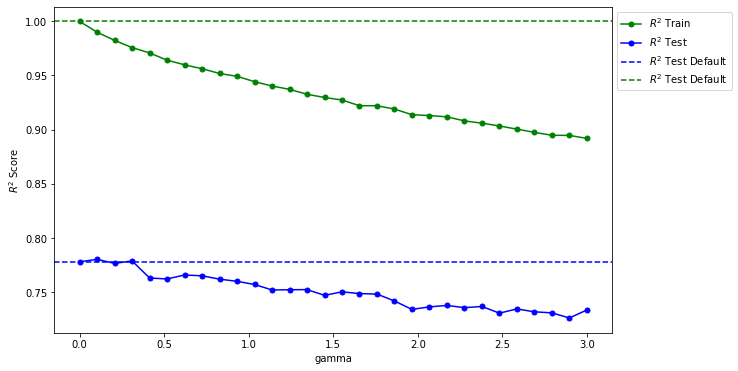


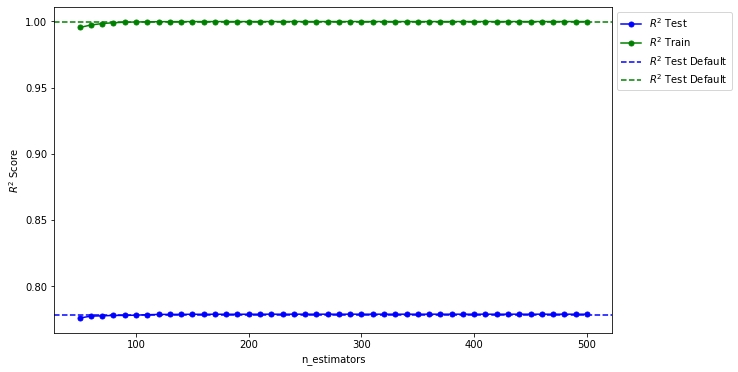


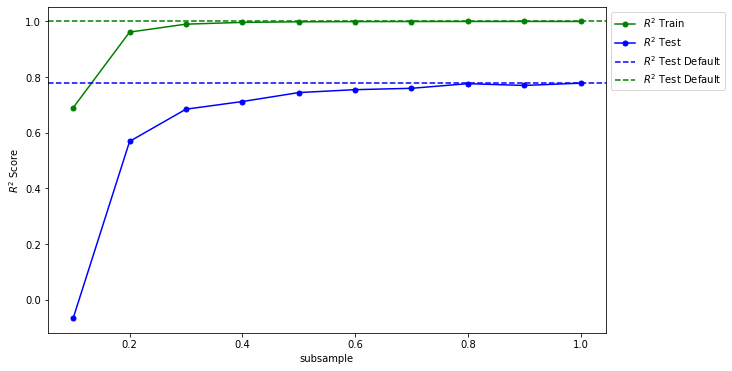


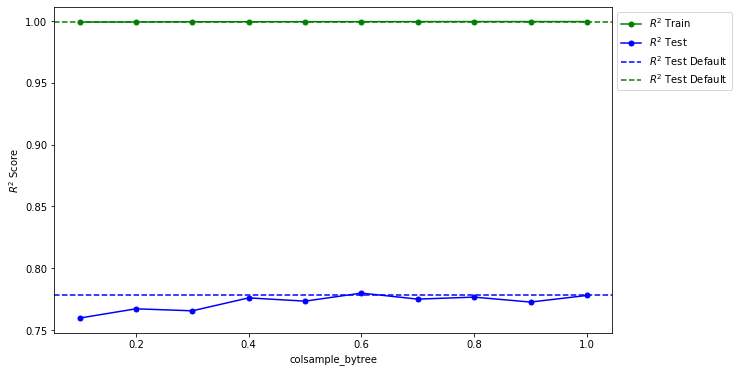


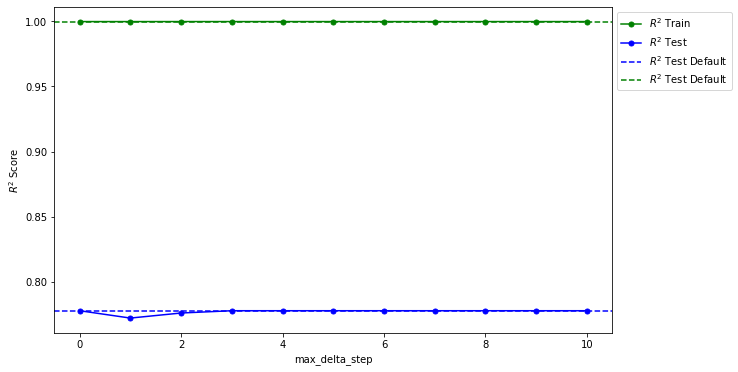


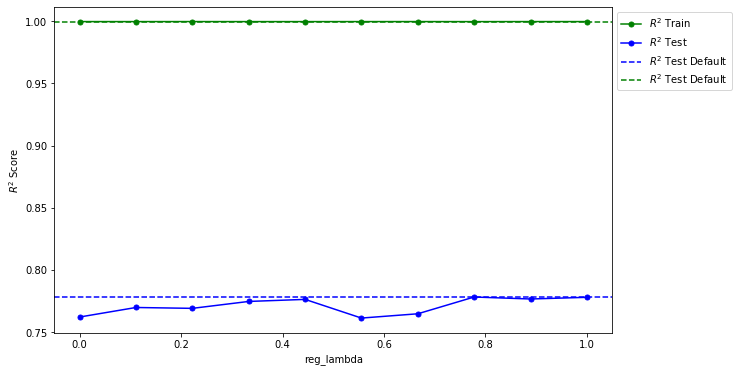


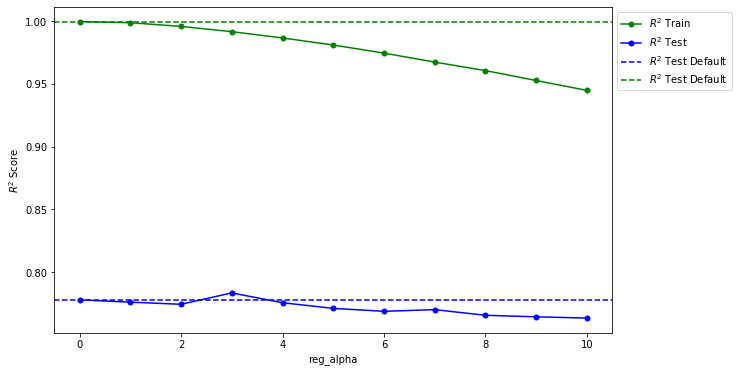


**Shallow neural network**

| **Parameter** | **Default** | **Start** | **End** | **Step size** | **Optimal** | **R^2^ _train_** | **R^2^_CV_** |
| --- | --- | --- | --- | --- | --- | --- | --- |
| Hidden layer nodes | 512 | 50 | 1000 | 20 | 400 | 0.953 | 0.793 |
| Dropout | 0 | 0 | 0.5 | 0.1 | 0.1 | 0.941 | 0.792 |
| Epochs | 100 | 50 | 500 | 10 | 100 | 0.953 | 0.792 |
| Batch size | 128 | 32 | 512 | x2 | 64 | 0.974 | 0.796 |
| Learning rate | 0.001 | 0.0001 | 0.003 | 0.0001 | 0.001 | 0.953 | 0.792 |


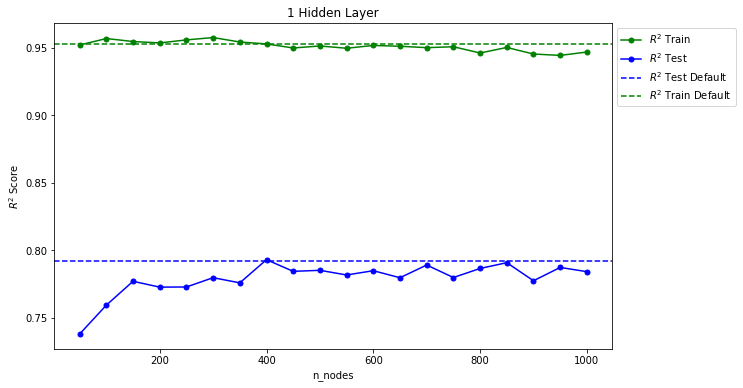


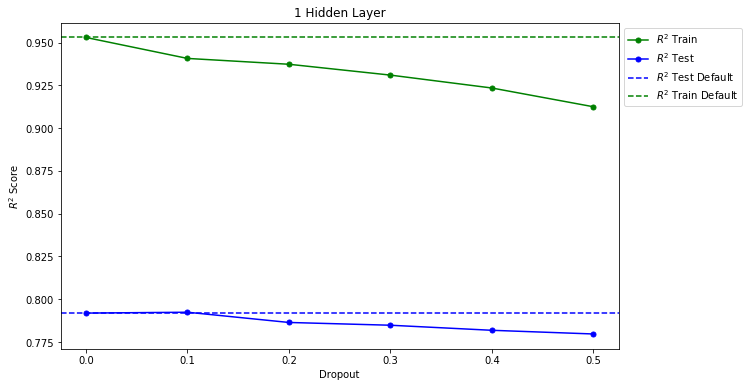


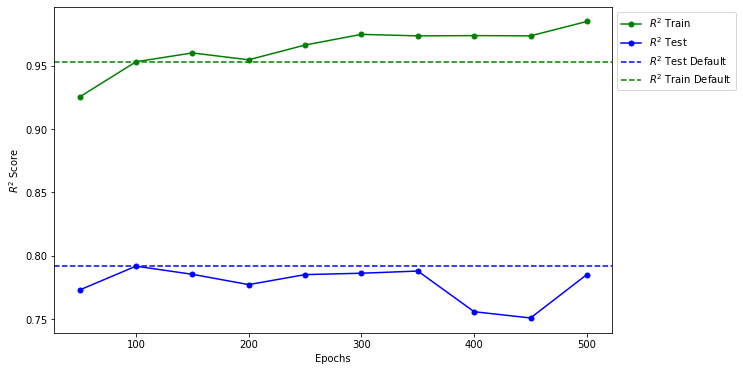


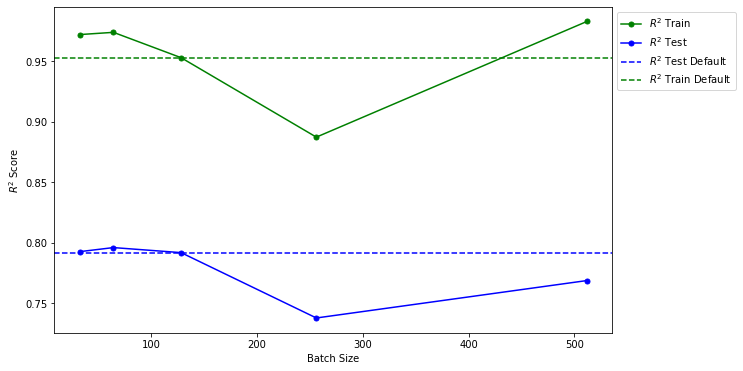


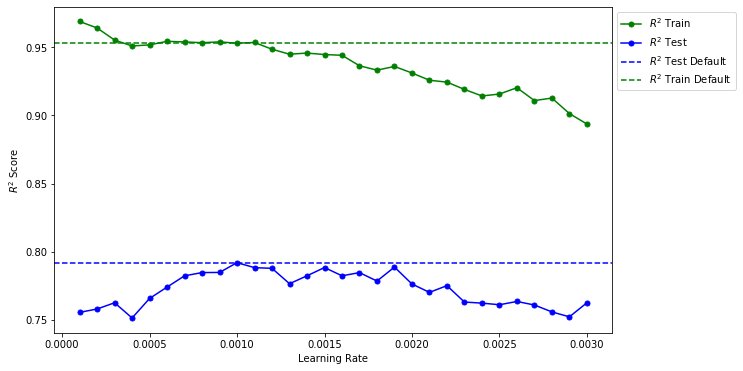


**Deep neural network**

| **Parameter*** | **Default** | **Start** | **End** | **Step size** | **Optimal** | **R^2^ _train_** | **R^2^_CV_** |
| --- | --- | --- | --- | --- | --- | --- | --- |
| Nodes per hidden layer (*n* = 2) | 512 | 50 | 1000 | 20 | 750 | 0.966 | 0.806 |
| Nodes per hidden layer (*n* = 3) | 512 | 50 | 1000 | 20 | 450 | 0.972 | 0.807 |
| Nodes per hidden layer (*n* = 4) | 512 | 50 | 1000 | 20 | 750 | 0.970 | 0.813 |
| Dropout, each hidden layer (*n* = 2) | 0 | 0 | 0.5 | 0.1 | 0.2 | 0.952 | 0.813 |
| Dropout, each hidden layer (*n* = 3) | 0 | 0 | 0.5 | 0.1 | 0.1 | 0.959 | 0.809 |
| Dropout, each hidden layer (*n* = 4) | 0 | 0 | 0.5 | 0.1 | 0 | 0.972 | 0.808 |
| Epochs | 100 | 50 | 500 | 10 | 100 | 0.953 | 0.792 |
| Batch size | 128 | 32 | 512 | x2 | 64 | 0.974 | 0.796 |
| Learning rate | 0.001 | 0.0001 | 0.003 | 0.0001 | 0.001 | 0.953 | 0.792 |

*n* = number of hidden layers

Rows highlighted in orange relate to aspects of network architecture (i.e., where number of hidden layers is greater than two) not considered for further evaluation.


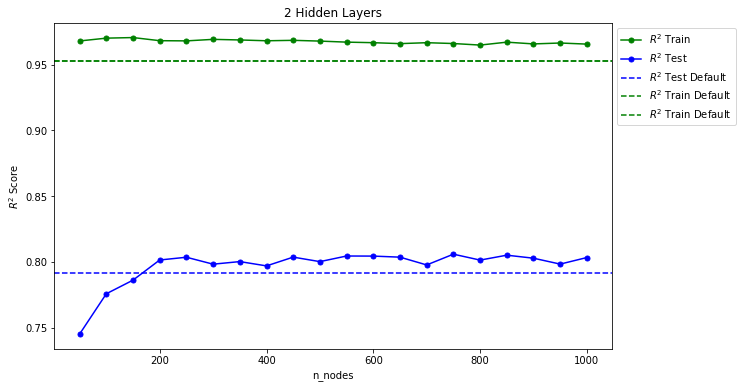


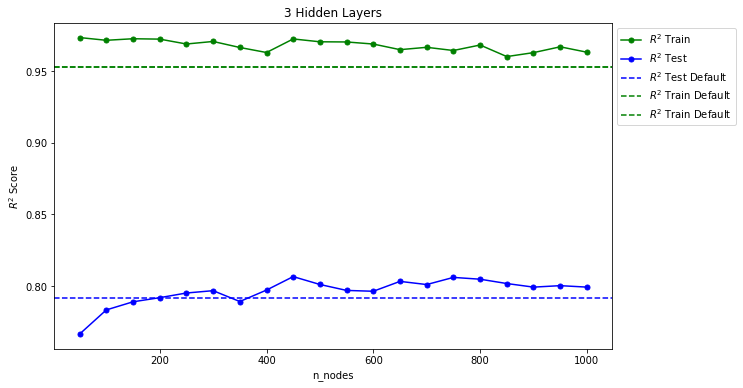


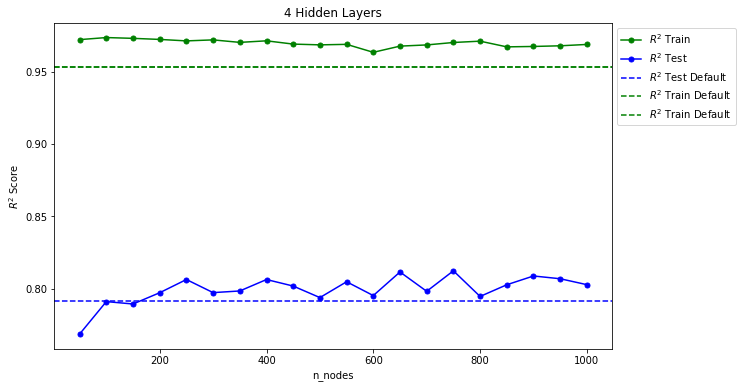


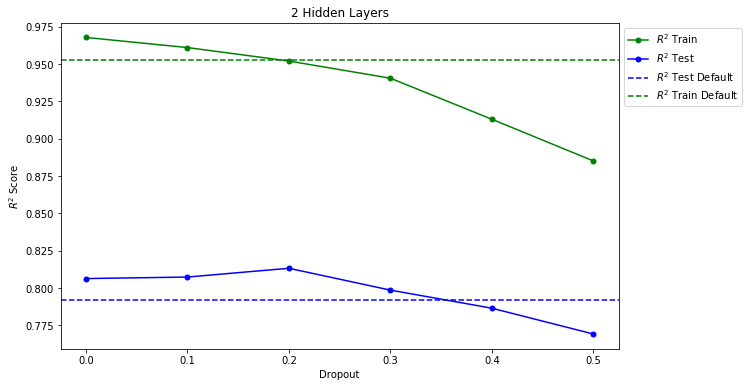


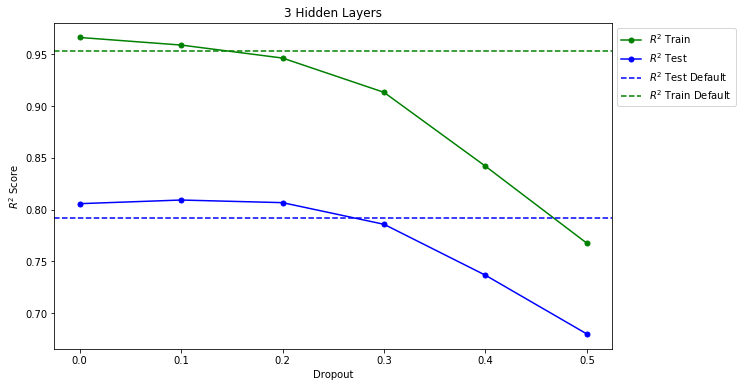


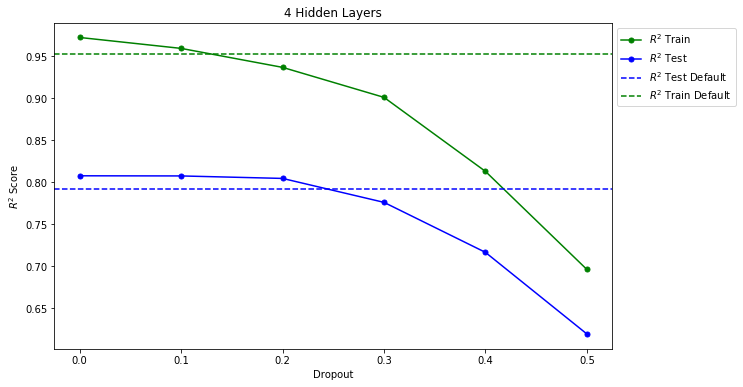


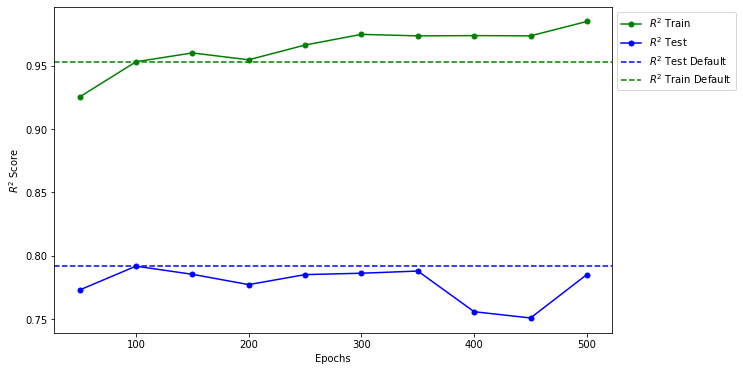


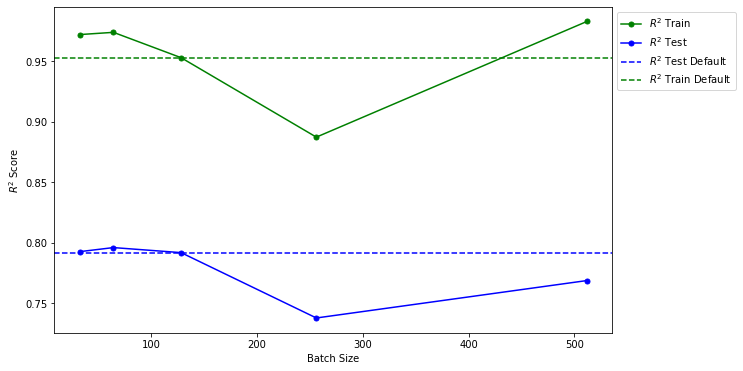


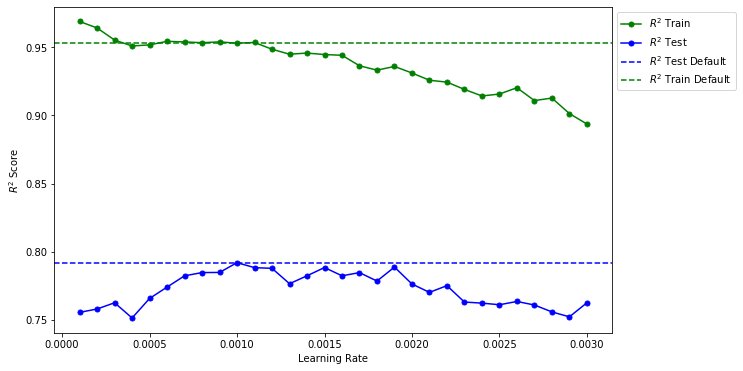

Supplement: S2 File — Hyperparameter optimisation (manual). Optimisation curves displayed within figures below outline influence upon the model performance metrics R2train and R2CV (k = 10), arising as a consequence of the incremental variation in value of a given hyperparameter over its defined range (with all others simultaneously held constant at their default quantities). (DOCX) [file pone.0282924.s002.docx]
