# Supplementary material for: Guidance for good practice in the application of machine learning in development of toxicological quantitative structure-activity relationships (QSARs)
Source: PLoS One. 2023 May 10;18(5):e0282924. doi: 10.1371/journal.pone.0282924 (PMC10171609; doi:10.1371/journal.pone.0282924)

**Supplementary Material 3.** Hyperparameter optimisation (randomised search)

Displayed within figures below are values of performance metrics R^2^_train_ and R^2^_CV_ (k = 10) relating to models generated through each trialled random hyperparameter combination.

The following legend is relatable to all:


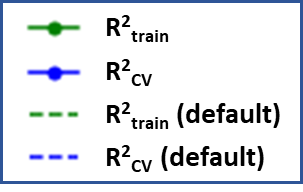


Tables provide a list those ranges examined with respect to each parameter, alongside step sizes separating quantities eligible for consideration within the search process. Further supplied are the corresponding values present within the optimally-performing sets.

**Random forest**

| **Parameter** | **Start** | **End** | **Step size** | **Optimal** |
| --- | --- | --- | --- | --- |
| max_depth | 10 | 30 | 1 | 30 |
| n_estimators | 100 | 500 | 10 | 490 |


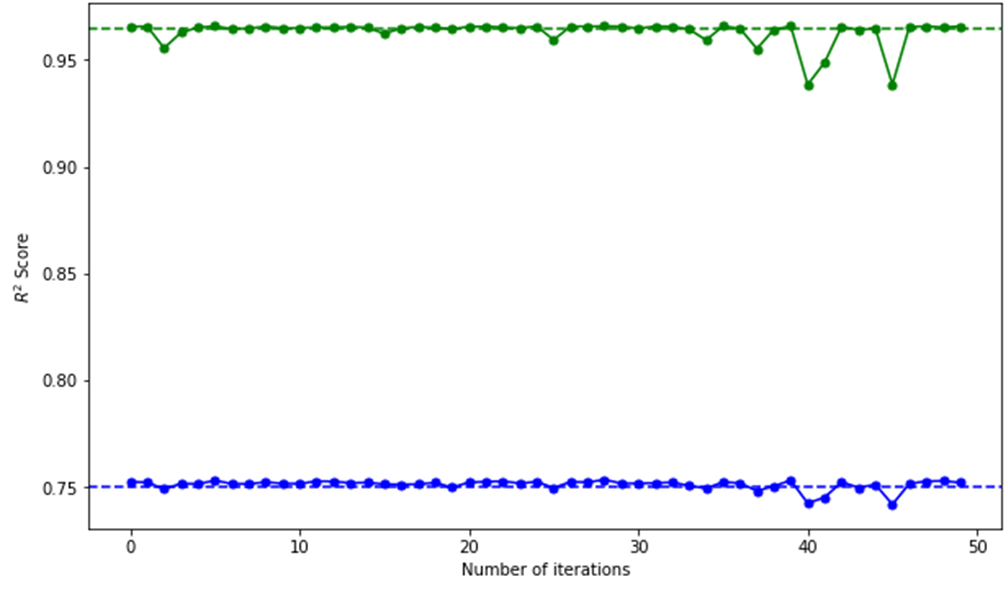


**Support vector machine**

| **Parameter** | **Start** | **End** | **Step size** | **Optimal** |
| --- | --- | --- | --- | --- |
| gamma | 0.0012 | 0.003 | 3.67E-05 | 0.0012 |
| C | 1 | 10 | 0.45 | 8.579 |
| epsilon | 0.001 | 0.02 | 0.001 | 0.018 |


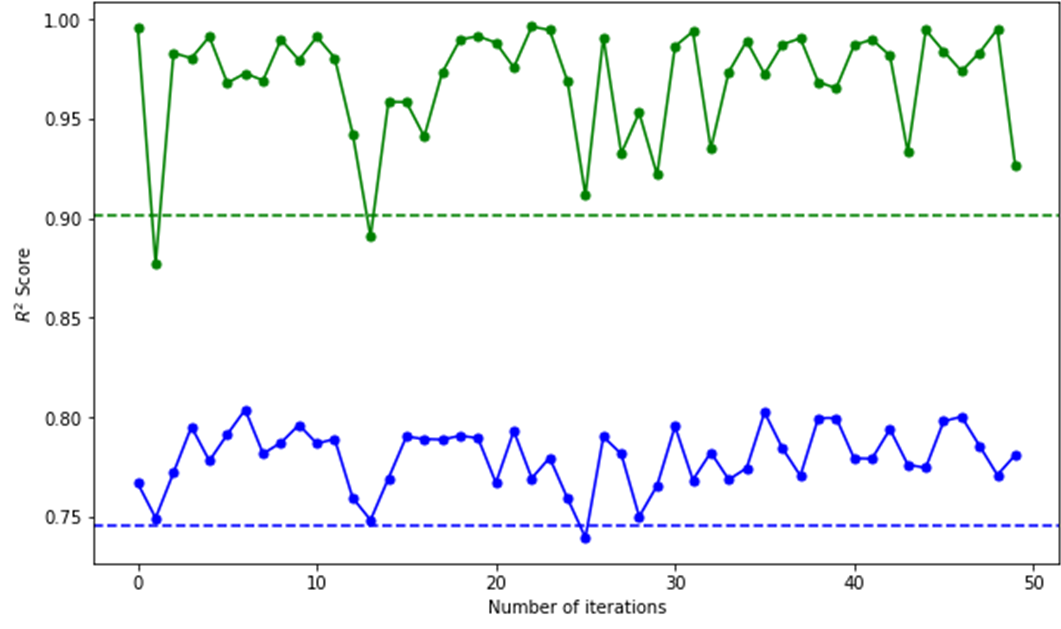


***k*-Nearest neighbours**

| **Parameter** | **Start** | **End** | **Step size** | **Optimal** |
| --- | --- | --- | --- | --- |
| n_neighbors | 1 | 15 | 1 | 3 |
| p | 1 | 3 | 1 | 1 |


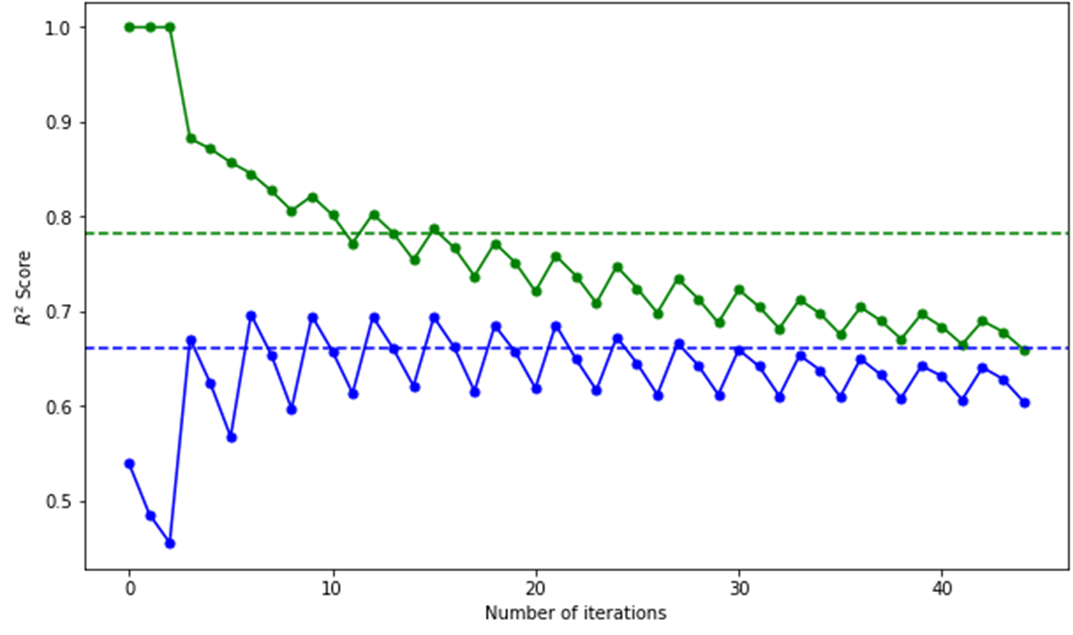


**Extreme gradient boosting**

| **Parameter** | **Start** | **End** | **Step size** | **Optimal** |
| --- | --- | --- | --- | --- |
| eta | 0.1 | 0.15 | 0.01 | 0.1 |
| gamma | 0 | 0.3 | 0.1 | 0.1 |
| max_depth | 2 | 8 | 1 | 4 |
| min_child_weight | 1 | 10 | 1 | 4 |
| subsample | 0.8 | 1 | 0.1 | 0.8 |
| colsample_bytree | 0.5 | 1 | 0.1 | 0.9 |
| n_estimators | 100 | 250 | 10 | 250 |


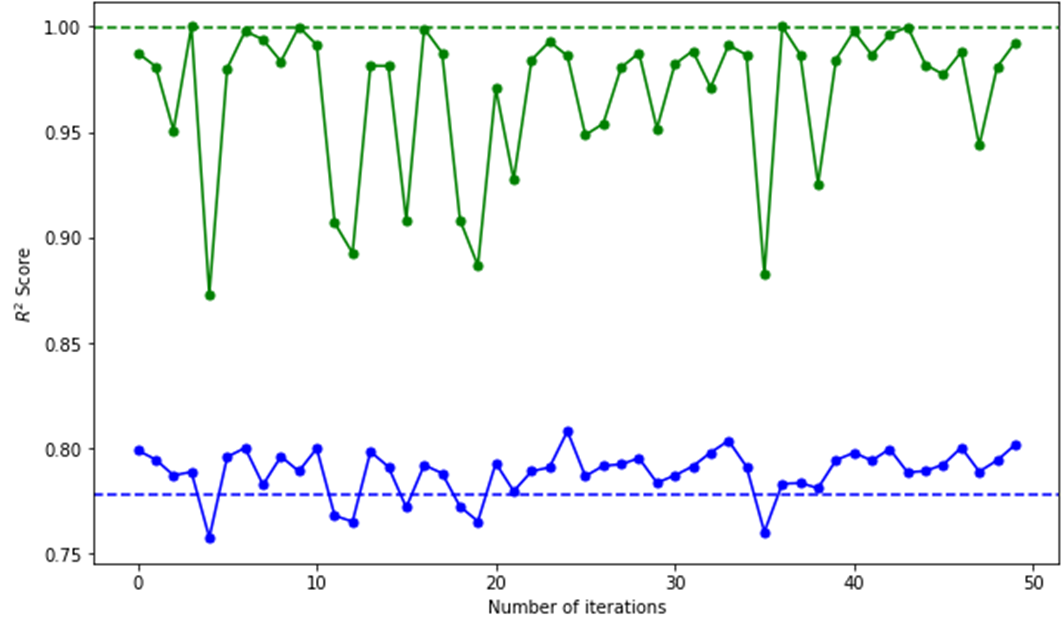


**Shallow neural network**

| **Parameter** | **Start** | **End** | **Step size** | **Optimal** |
| --- | --- | --- | --- | --- |
| neurons | 50 | 1000 | 50 | 550 |
| dropout_rate | 0 | 0.5 | 0.1 | 0.2 |
| learn_rate | 0.0001 | 0.001 | 0.0001 | 0.0003 |
| epochs | 50 | 500 | 50 | 250 |
| batch_size | 32 | 512 | x2 | 64 |


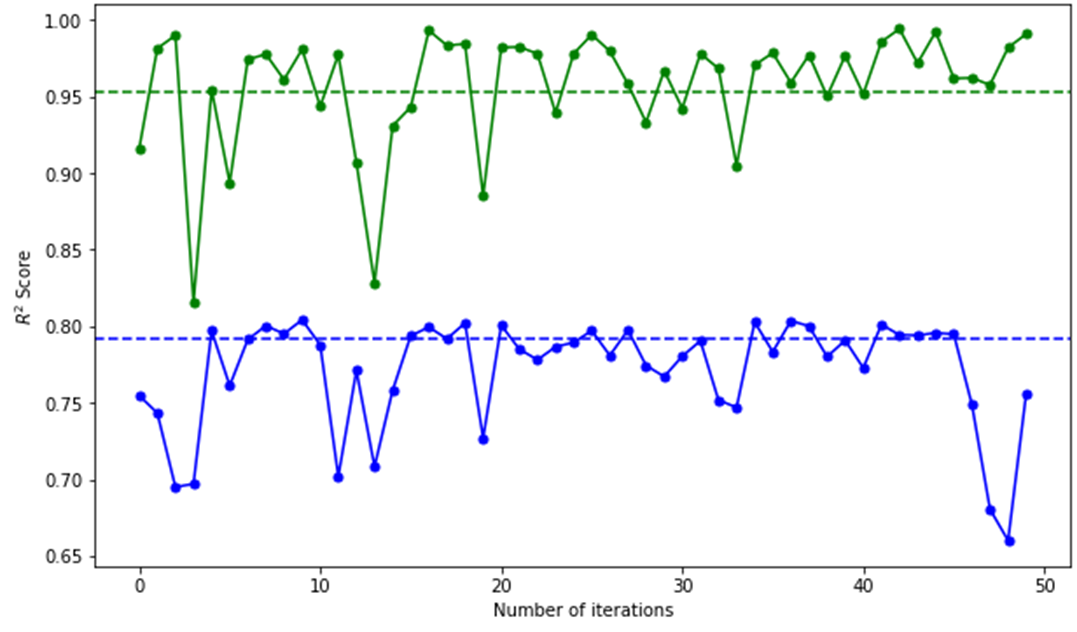


**Deep neural network**

| **Parameter** | **Start** | **End** | **Step size** | **Optimal** |
| --- | --- | --- | --- | --- |
| neurons | 50 | 1000 | 50 | 650 |
| neurons_l2 | 50 | 1000 | 50 | 50 |
| dropout_rate | 0 | 0.5 | 0.1 | 0.3 |
| dropout_rate_l2 | 0 | 0.5 | 0.1 | 0.4 |
| learn_rate | 0.0001 | 0.001 | 0.0001 | 0.0003 |
| epochs | 50 | 500 | 50 | 500 |
| batch_size | 32 | 512 | x2 | 32 |


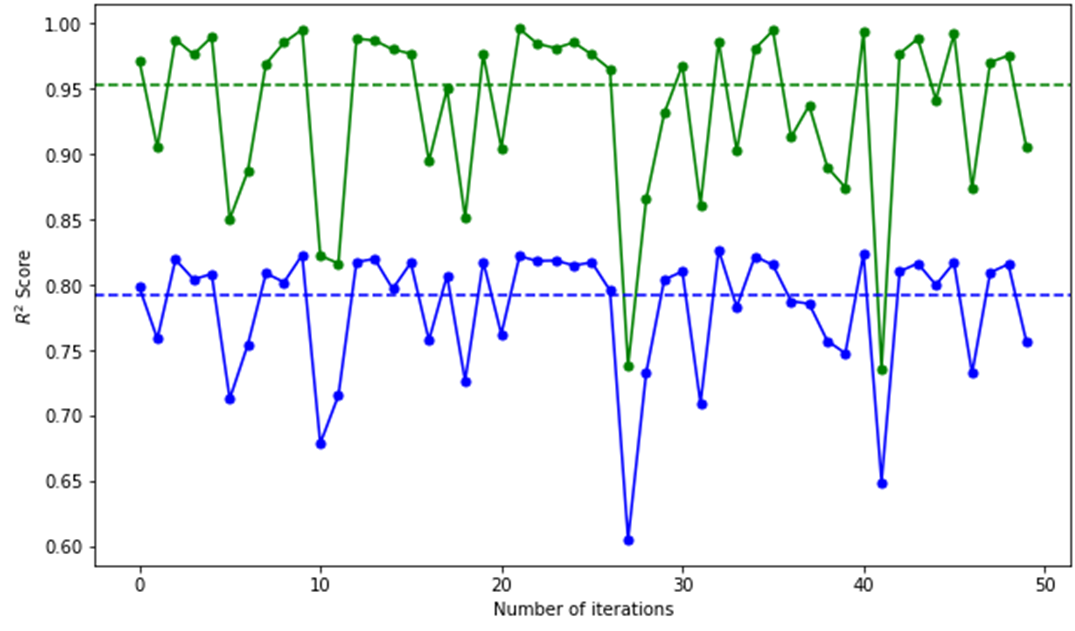

Supplement: S3 File — Hyperparameter optimisation (randomised search). Displayed within figures below are values of performance metrics R2train and R2CV (k = 10) relating to models generated through each trialled random hyperparameter combination. (DOCX) [file pone.0282924.s003.docx]
