# Supplementary material for: Guidance for good practice in the application of machine learning in development of toxicological quantitative structure-activity relationships (QSARs)
Source: PLoS One. 2023 May 10;18(5):e0282924. doi: 10.1371/journal.pone.0282924 (PMC10171609; doi:10.1371/journal.pone.0282924)

**Supplementary Material 4.** Hyperparameter optimisation (Bayesian)

Figures below illustrate aspects of the iterative Bayesian optimisation procedure enacted upon each model, courtesy of Optuna.

**Optimisation history plot:** Performance of model (quantified in terms of “objective value”) as constructed using hyperparameter combinations identified at respective iteration step.

**Slice plot:** Variation in values of individual parameters adopted with each stage of iteration, alongside influence upon overall model performance.

**Contour plot:** Overview of model performance, inferred across all possible combinations of values between hyperparameter pair (within range).

**Hyperparameter importances:** Relative influence of individual hyperparameter within the driving of the optimisation process.

Tables provide a list those ranges examined with respect to each parameter, alongside identities of the distribution functions adopted for purposes of generating updated quantities applicable to the search. Further supplied are the corresponding values present within the optimally-performing sets.

**Random forest**

| **Parameter** | **Start** | **End** | **Suggest** | **Optimal** |
| --- | --- | --- | --- | --- |
| max_depth | 10 | 30 | int | 27 |
| n_estimators | 100 | 500 | int | 499 |


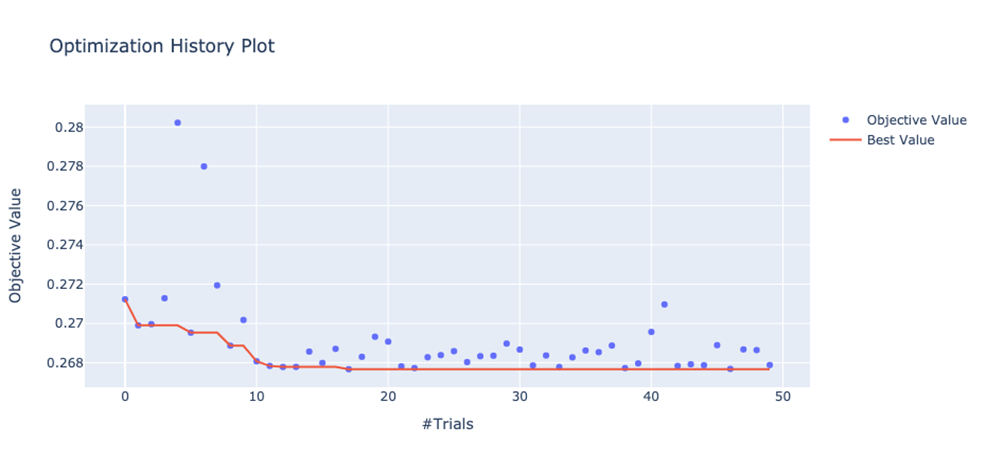

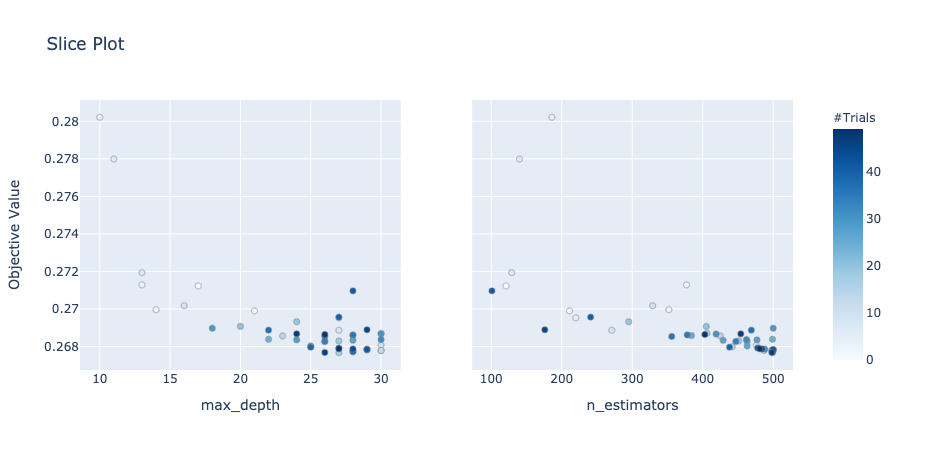


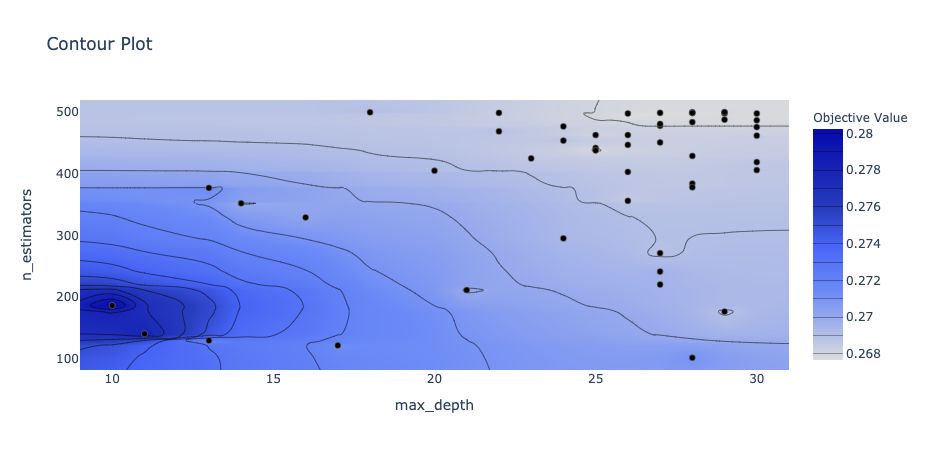


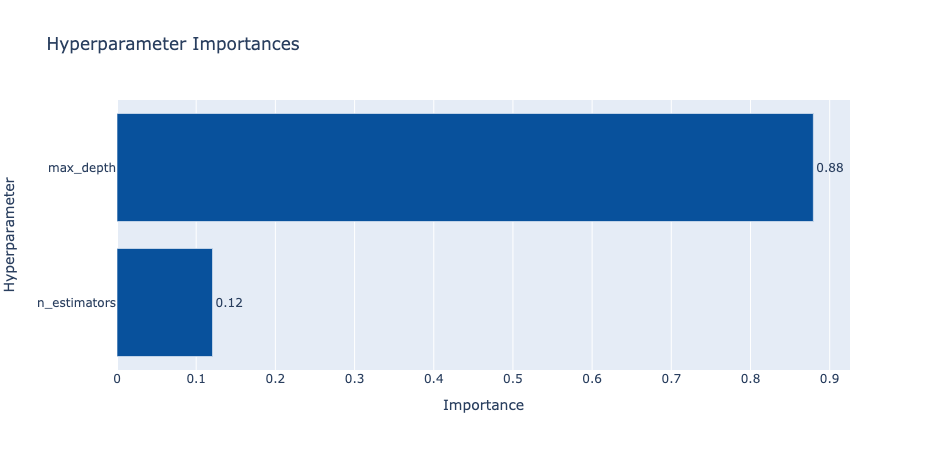


**Support vector machine**

| **Parameter** | **Start** | **End** | **Suggest** | **Optimal** |
| --- | --- | --- | --- | --- |
| gamma | 0.0012 | 0.003 | loguniform | 0.0012 |
| C | 1 | 20 | loguniform | 9.938 |
| epsilon | 0.001 | 0.02 | loguniform | 0.009 |


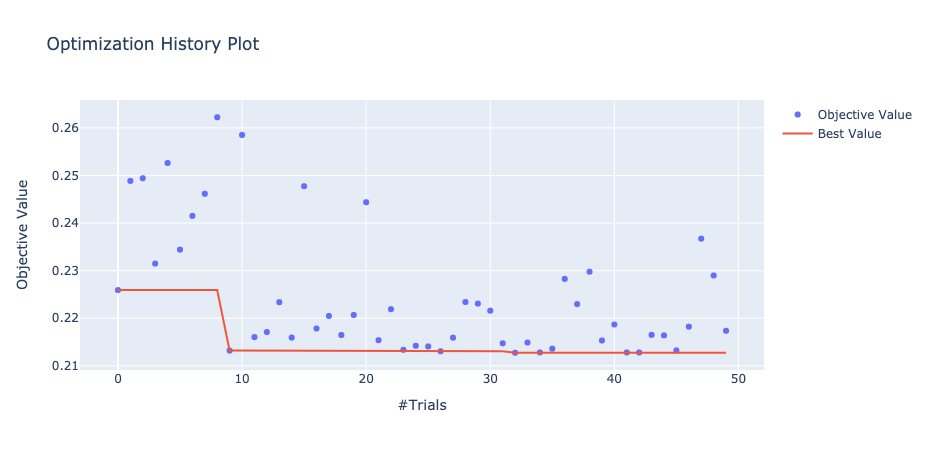

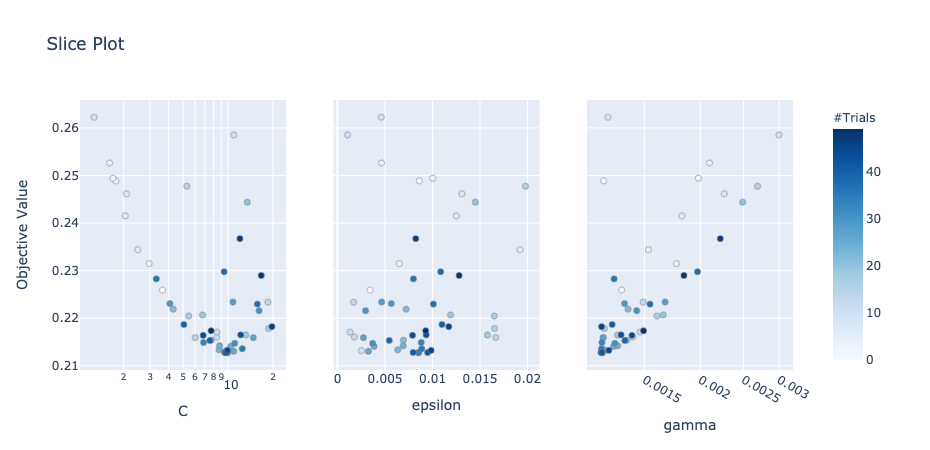


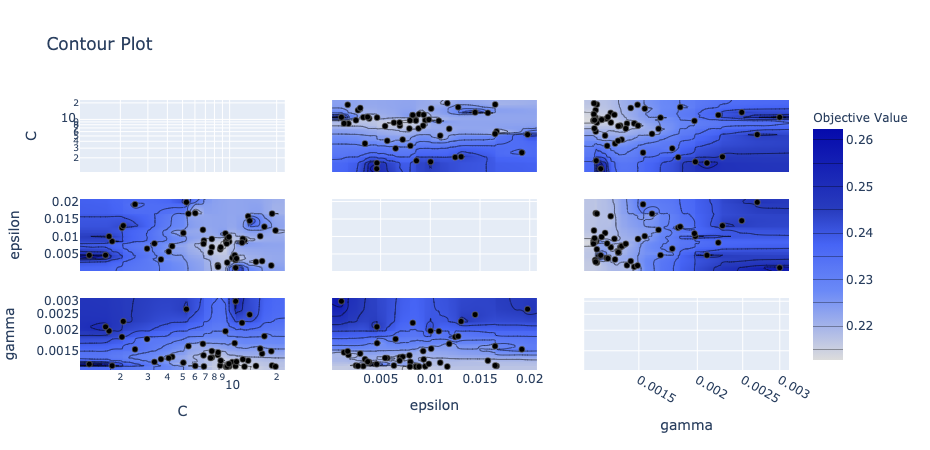


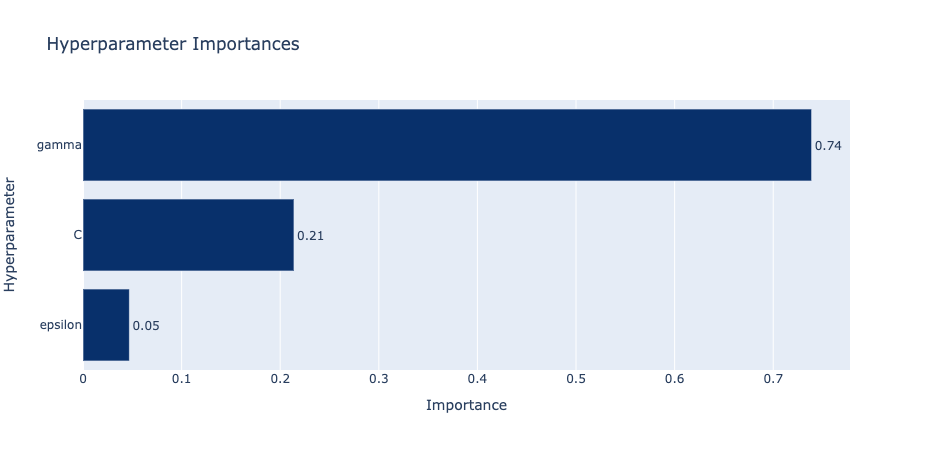


***k*-Nearest neighbours**

| **Parameter** | **Start** | **End** | **Suggest** | **Optimal** |
| --- | --- | --- | --- | --- |
| n_neighbors | 1 | 15 | int | 3 |
| p | 1 | 3 | int | 1 |


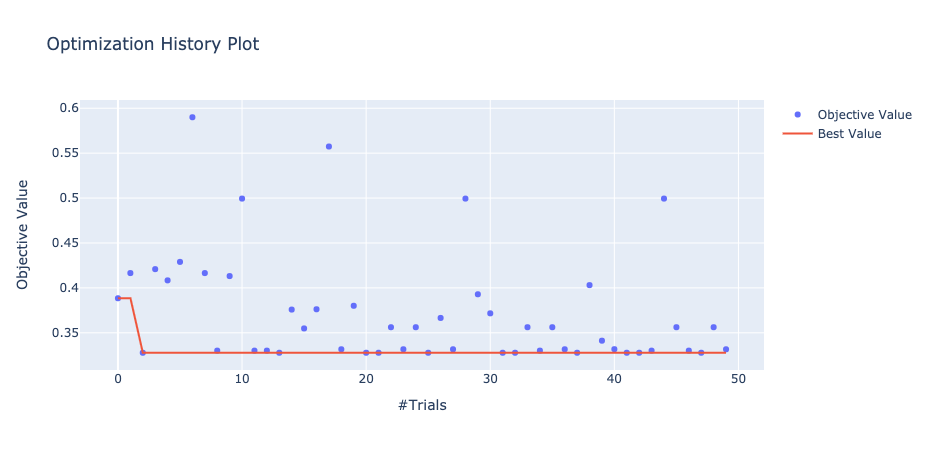

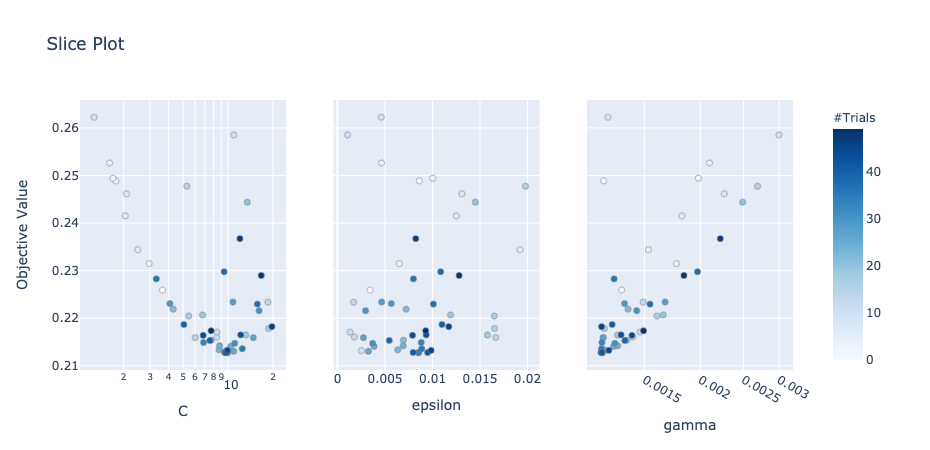


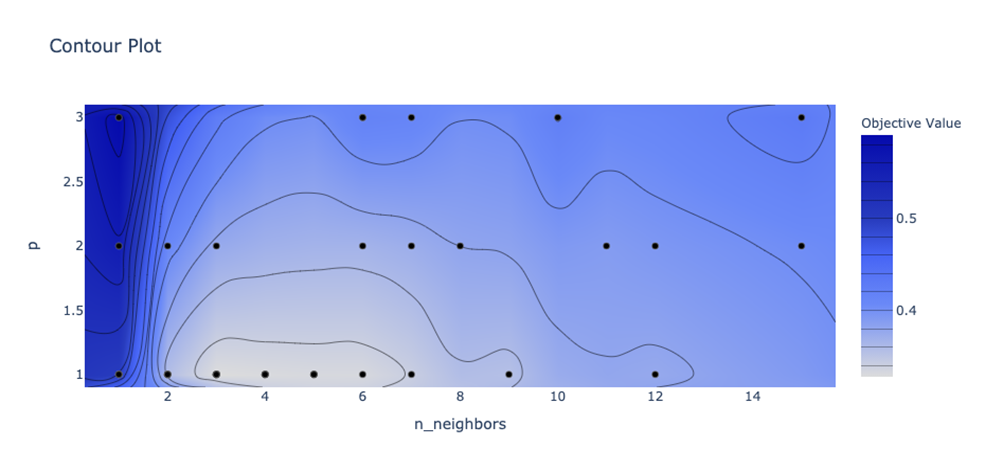


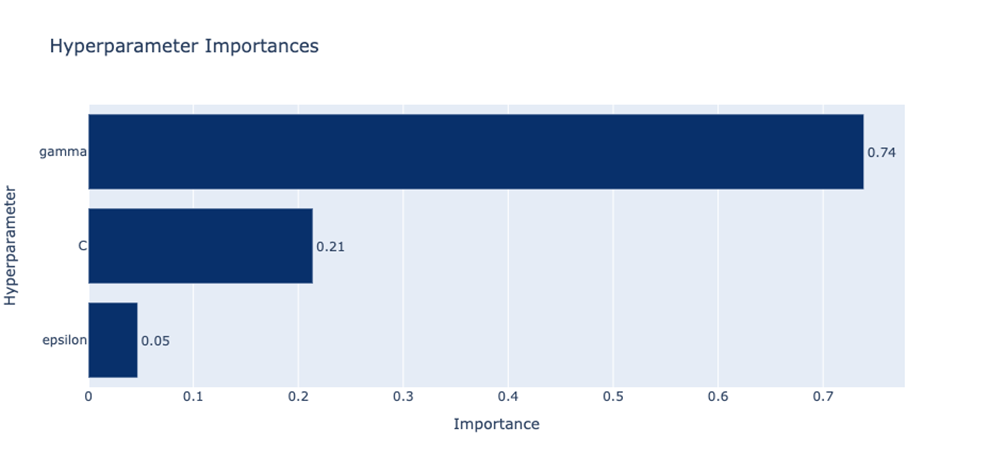


**Extreme gradient boosting**

| **Parameter** | **Start** | **End** | **Suggest** | **Optimal** |
| --- | --- | --- | --- | --- |
| eta | 0.1 | 0.15 | loguniform | 0.103 |
| gamma | 0 | 0.3 | uniform | 0.00145 |
| max_depth | 2 | 8 | int | 5 |
| min_child_weight | 1 | 10 | int | 2 |
| subsample | 0.8 | 1 | uniform | 0.816 |
| colsample_bytree | 0.5 | 1 | uniform | 0.962 |
| n_estimators | 100 | 250 | int | 205 |


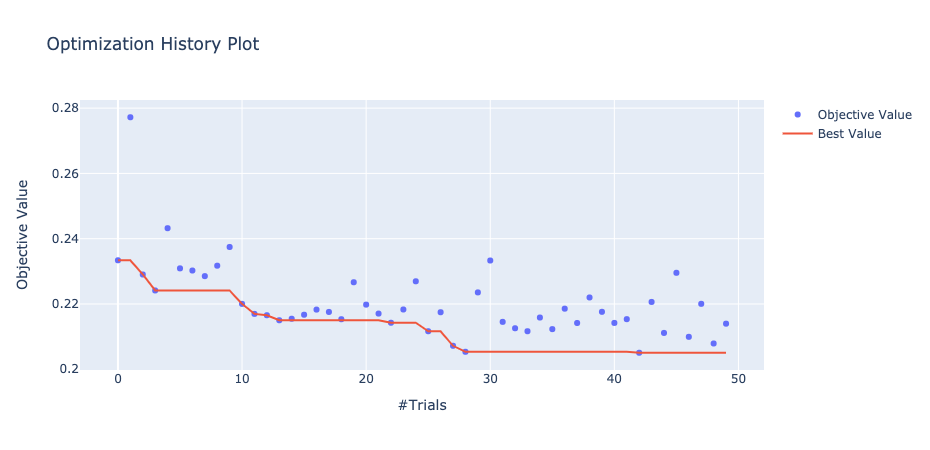

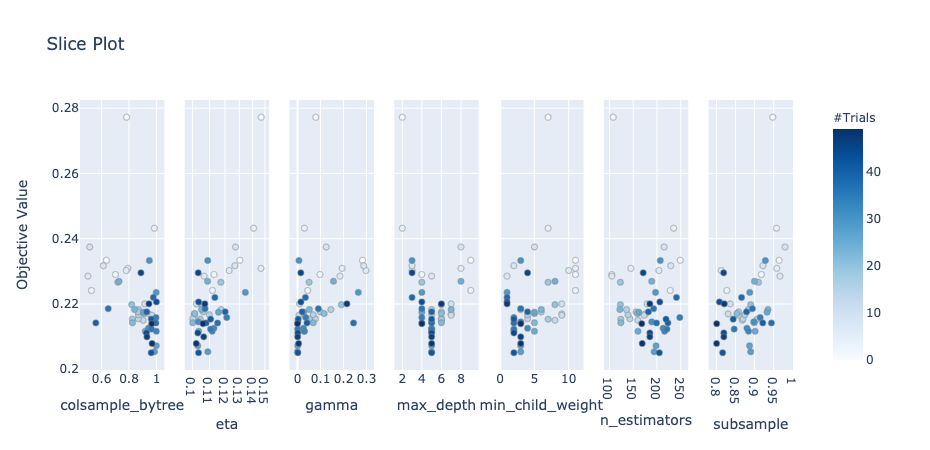


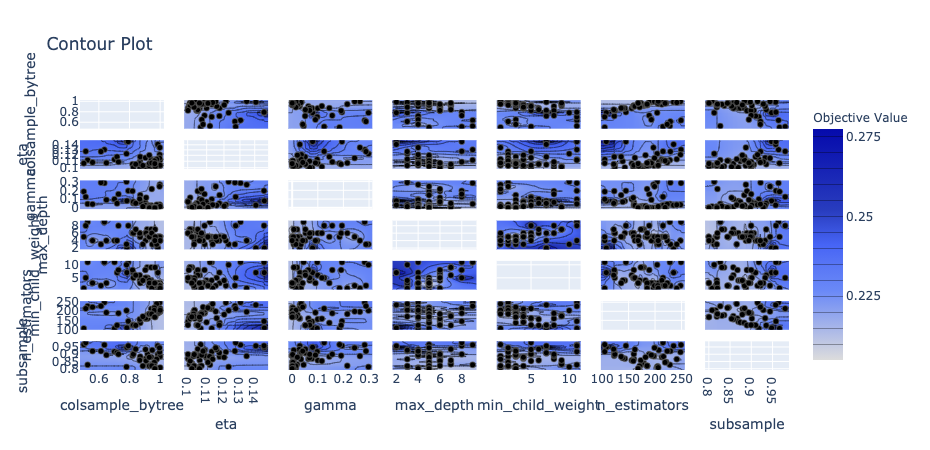


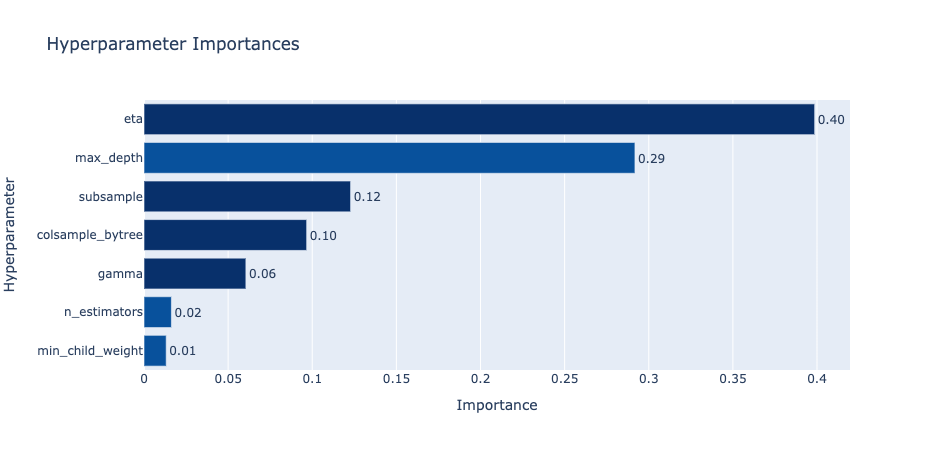


**Shallow neural network**

| **Parameter** | **Start** | **End** | **Suggest** | **Optimal** |
| --- | --- | --- | --- | --- |
| neurons | 50 | 1000 | int | 601 |
| dropout_rate | 0 | 0.5 | float | 0.444 |
| learn_rate | 0.0001 | 0.001 | loguniform | 3.76E-04 |
| epochs | 50 | 500 | int | 236 |
| batch_size | 10 | 500 | int | 197 |


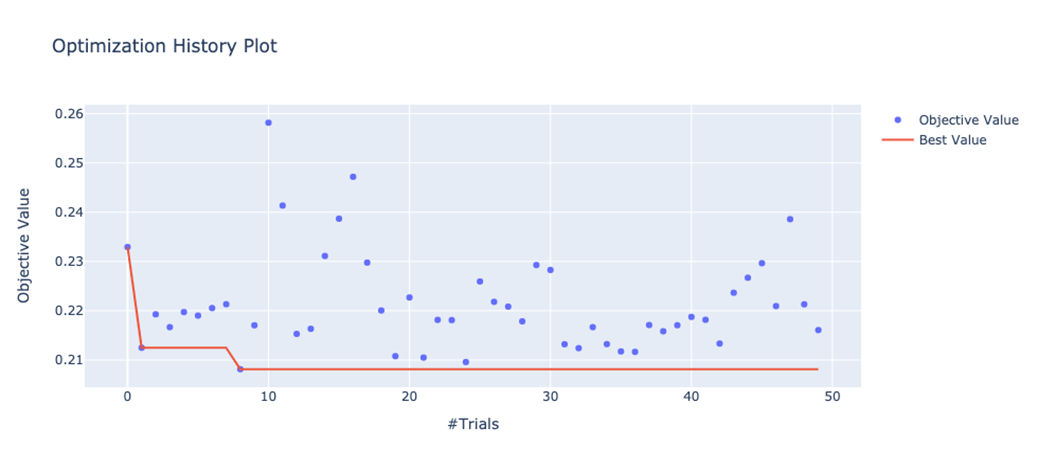

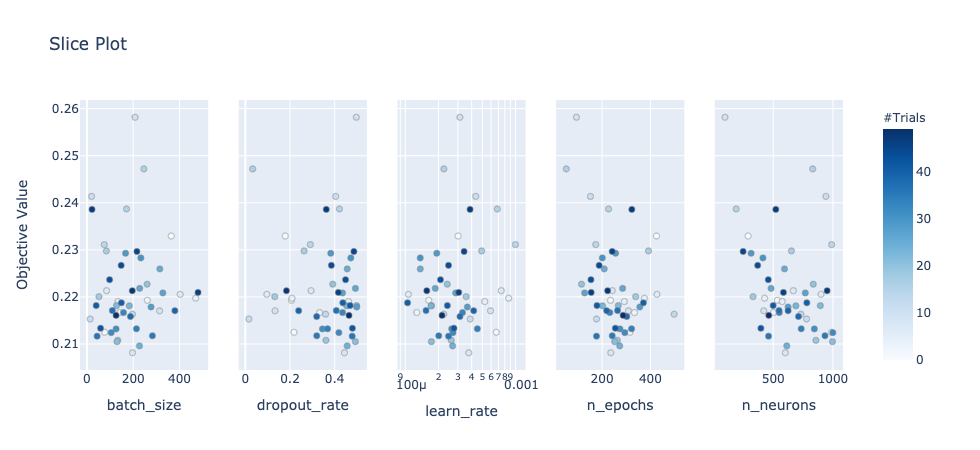


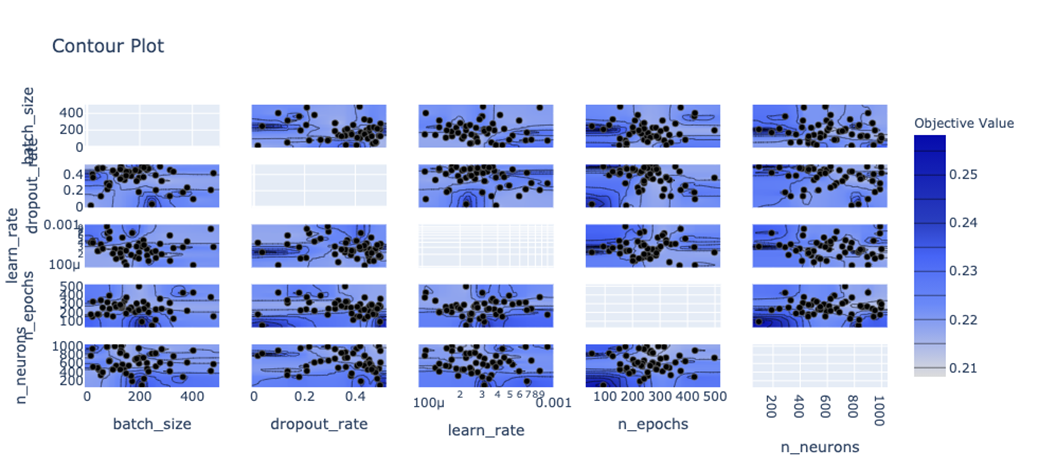


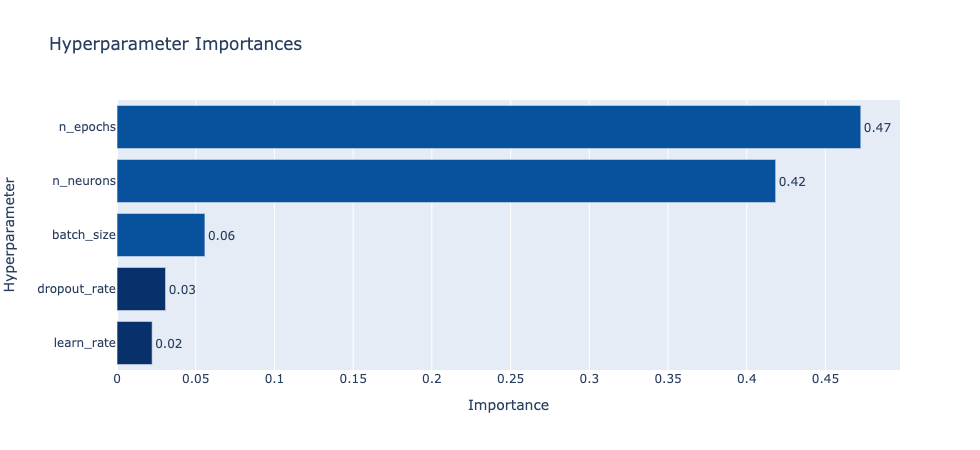


**Deep neural network**

| **Parameter** | **Start** | **End** | **Suggest** | **Optimal** |
| --- | --- | --- | --- | --- |
| neurons | 50 | 1000 | int | 944 |
| neurons_l2 | 50 | 1000 | int | 784 |
| dropout_rate | 0 | 0.5 | float | 0.161 |
| dropout_rate_l2 | 0 | 0.5 | float | 0.494 |
| learn_rate | 0.0001 | 0.001 | loguniform | 3.21E-04 |
| epochs | 50 | 500 | int | 498 |
| batch_size | 10 | 500 | int | 75 |


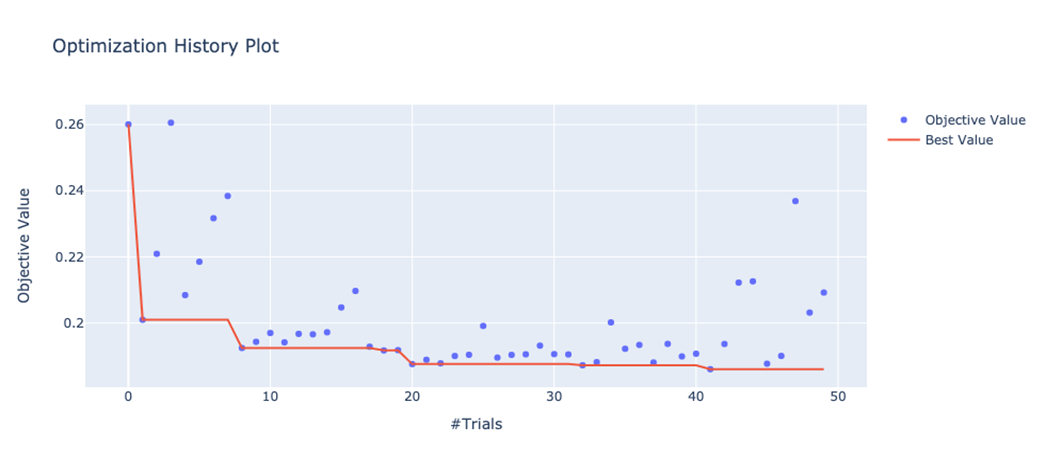

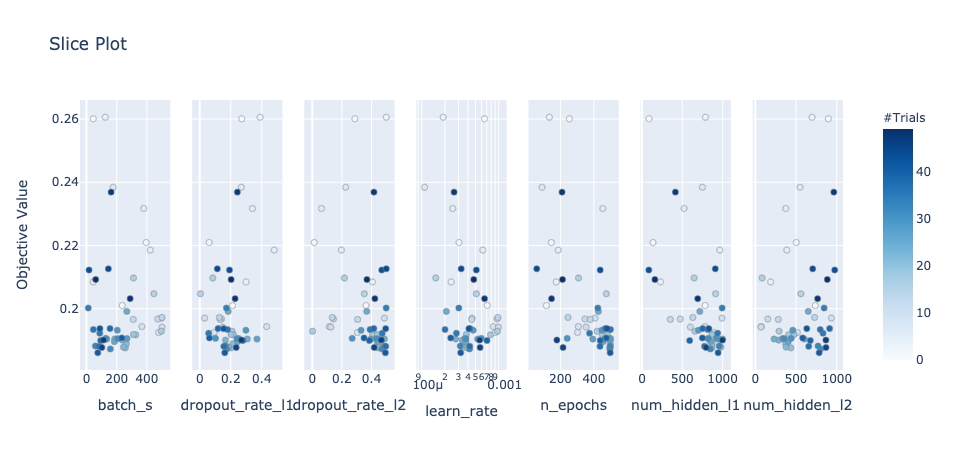


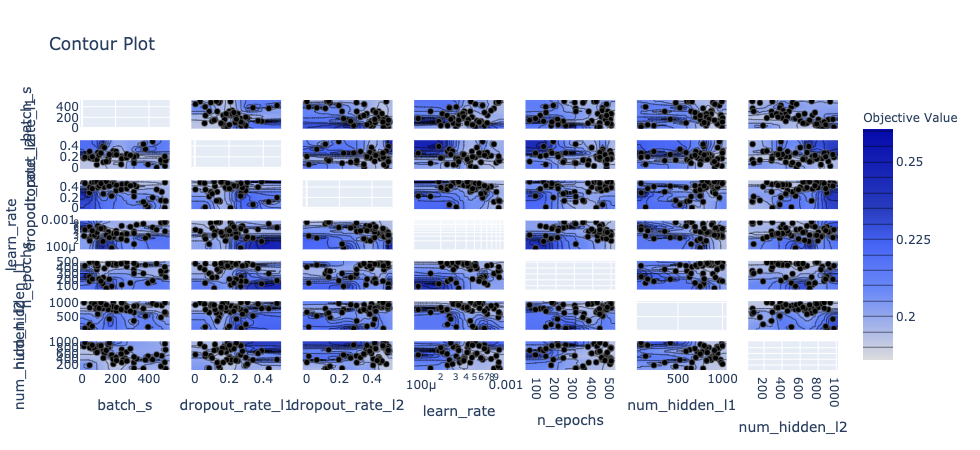


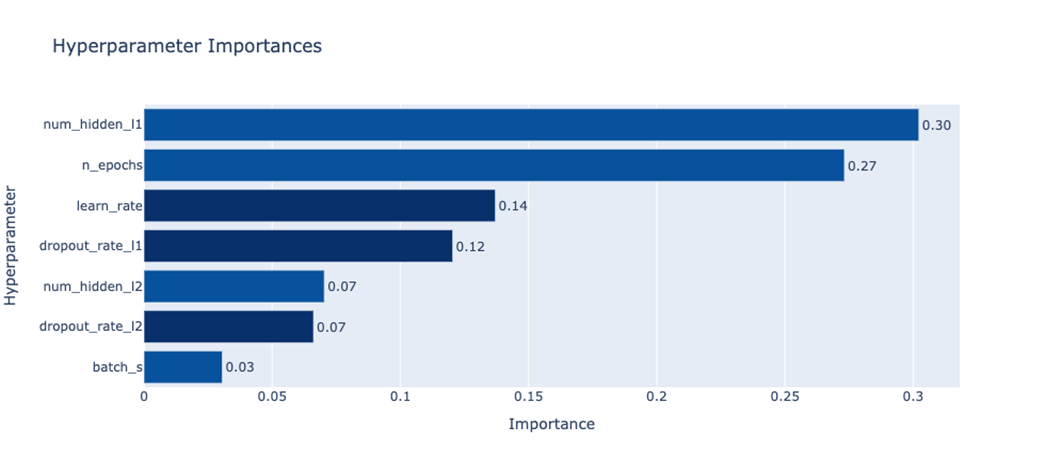

Supplement: S4 File — Hyperparameter optimisation (Bayesian). Figures below illustrate aspects of the iterative Bayesian optimisation procedure enacted upon each model, courtesy of Optuna. (DOCX) [file pone.0282924.s004.docx]
