# Supplementary material for: Guidance for good practice in the application of machine learning in development of toxicological quantitative structure-activity relationships (QSARs)
Source: PLoS One. 2023 May 10;18(5):e0282924. doi: 10.1371/journal.pone.0282924 (PMC10171609; doi:10.1371/journal.pone.0282924)
Supplement: S5 File — SHAP-determined absolute global feature importances relating to each model, as trained upon T. pyriformis TH_90 and TH_50 data subsets through adoption of Optuna-derived hyperparameter sets. (DOCX) [file pone.0282924.s005.docx]

**Supplementary Material 5**

SHAP-determined **a**bsolute global feature importances relating to each model, as trained upon *T. pyriformis* TH_90 and TH_50 data subsets through adoption of Optuna-derived hyperparameter sets.

**Random forest**

TH_90 data subset


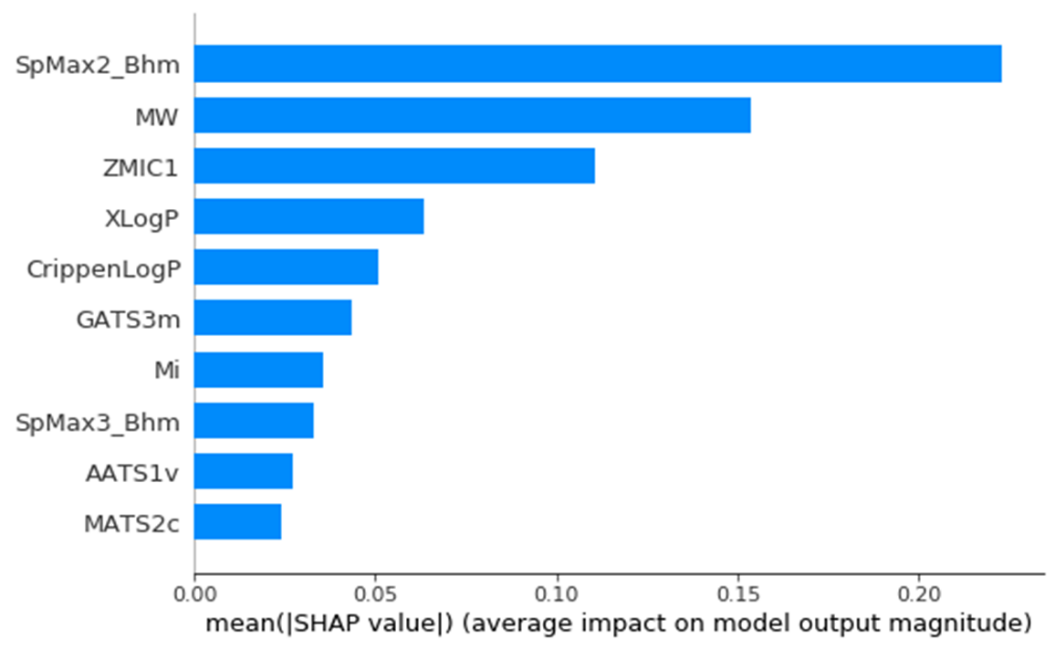


TH_50 data subset


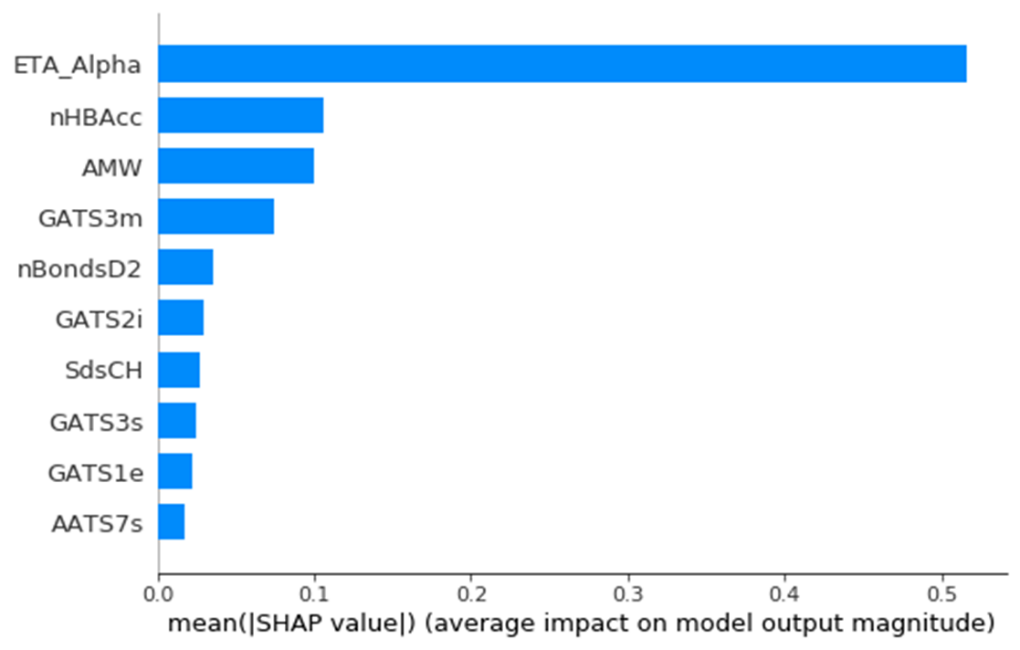


**Support vector machine**

TH_90 data subset

**
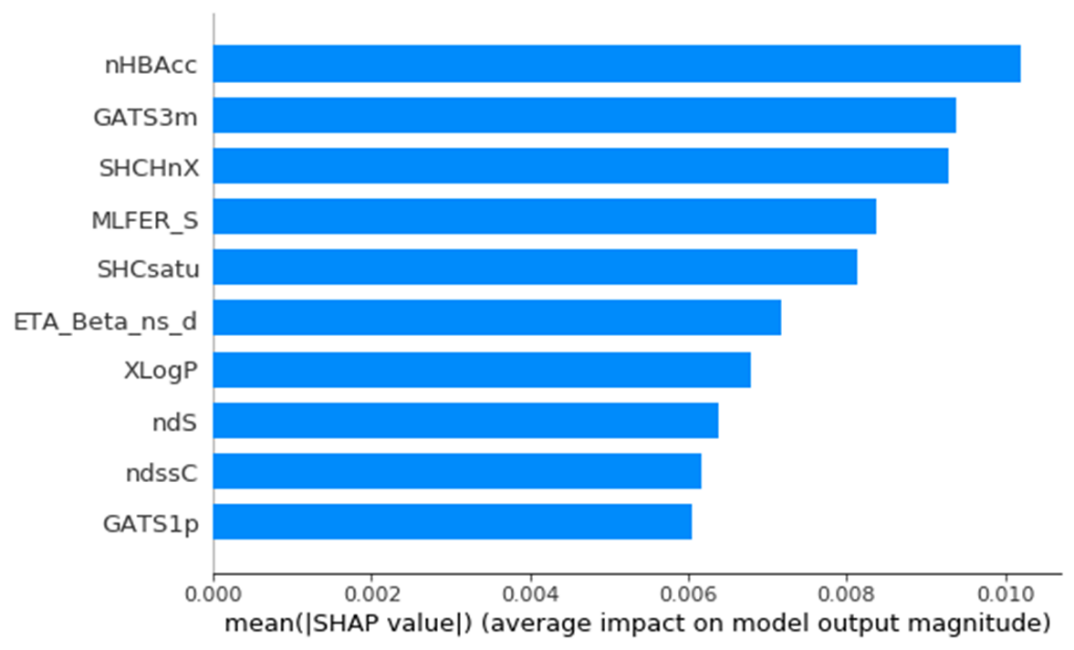
**

TH_50 data subset

**
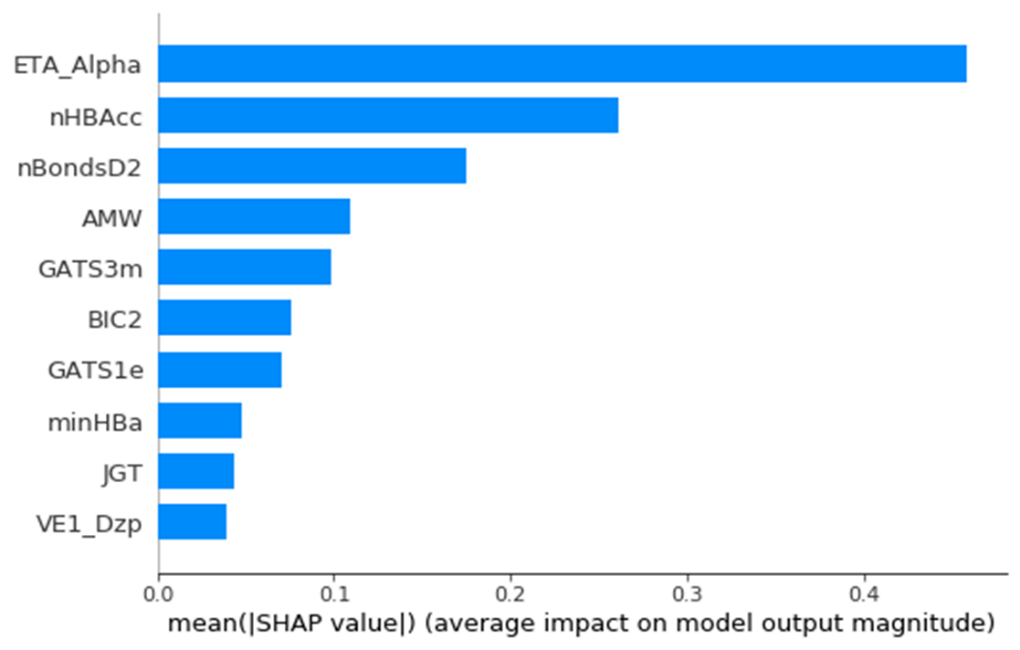
**

***k*-Nearest neighbours**

TH_90 data subset

**
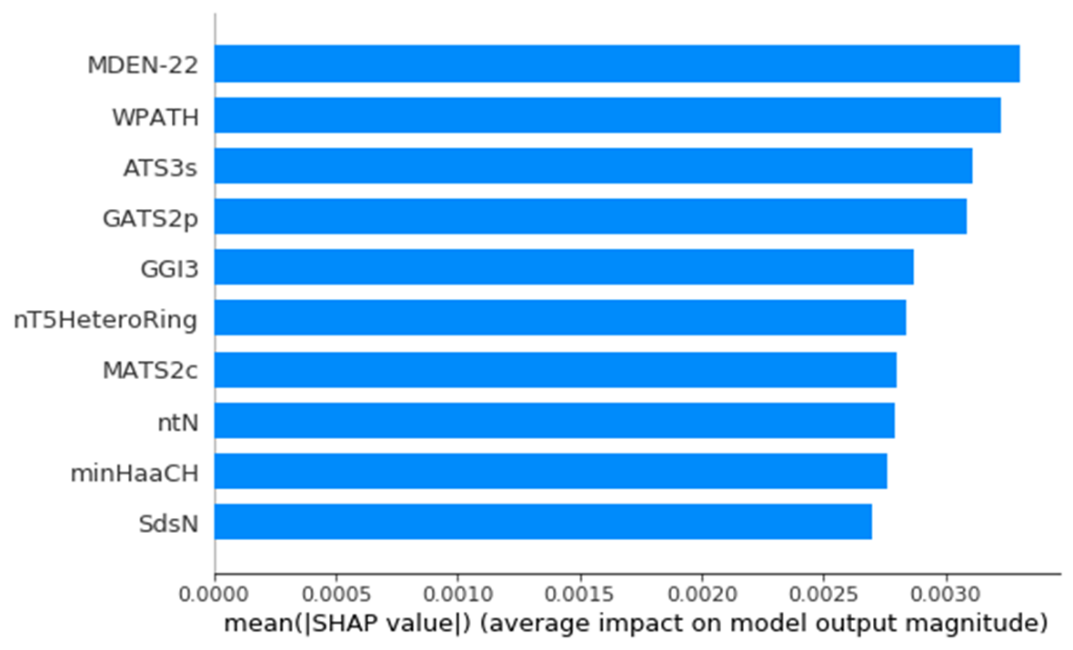
**

TH_50 data subset


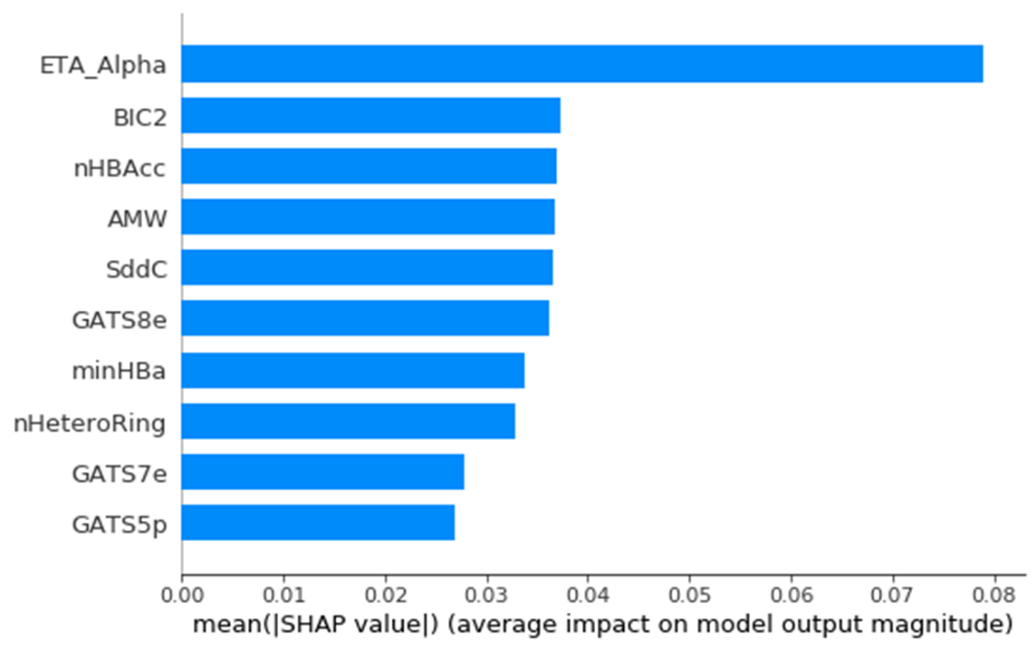


**Extreme gradient boosting**

TH_90 data subset

**
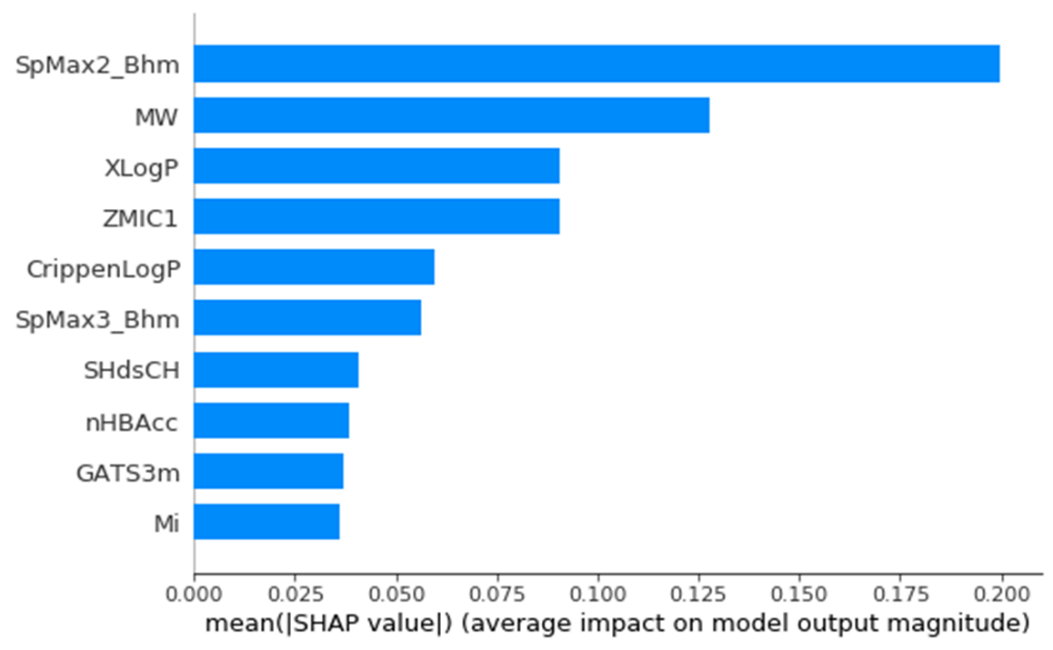
**

TH_50 data subset

**
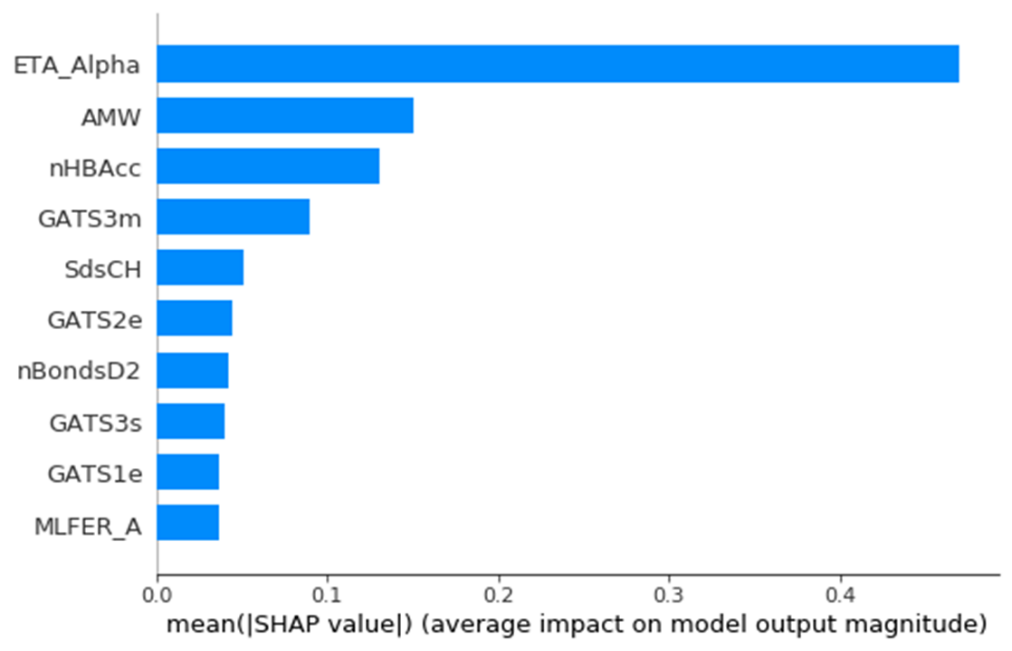
**

**Shallow neural network**

TH_90 data subset


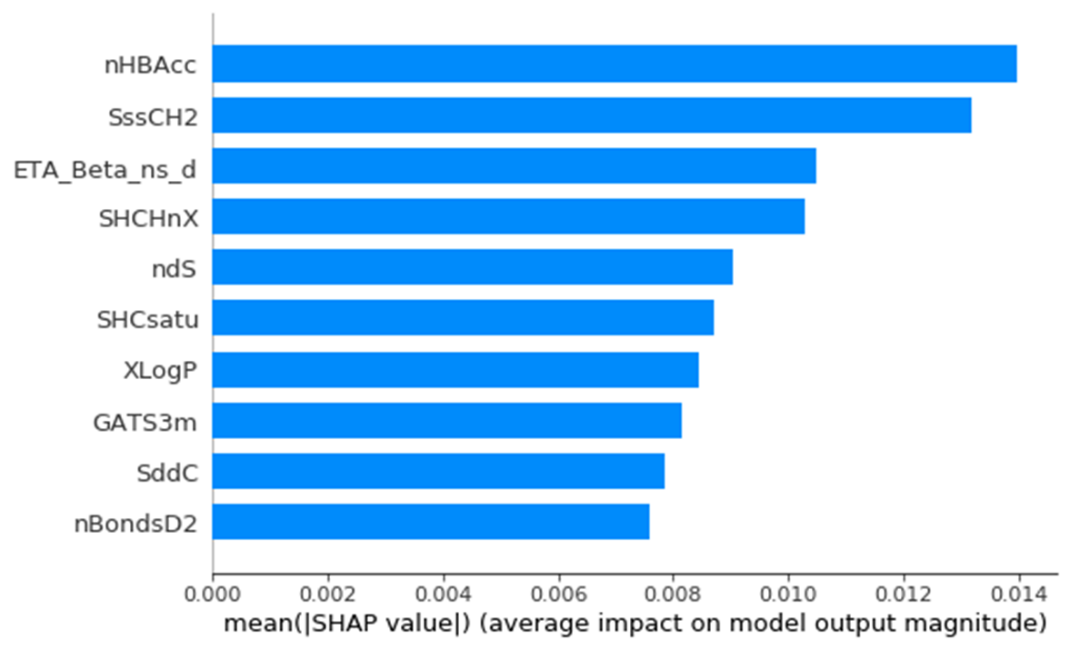


TH_50 data subset


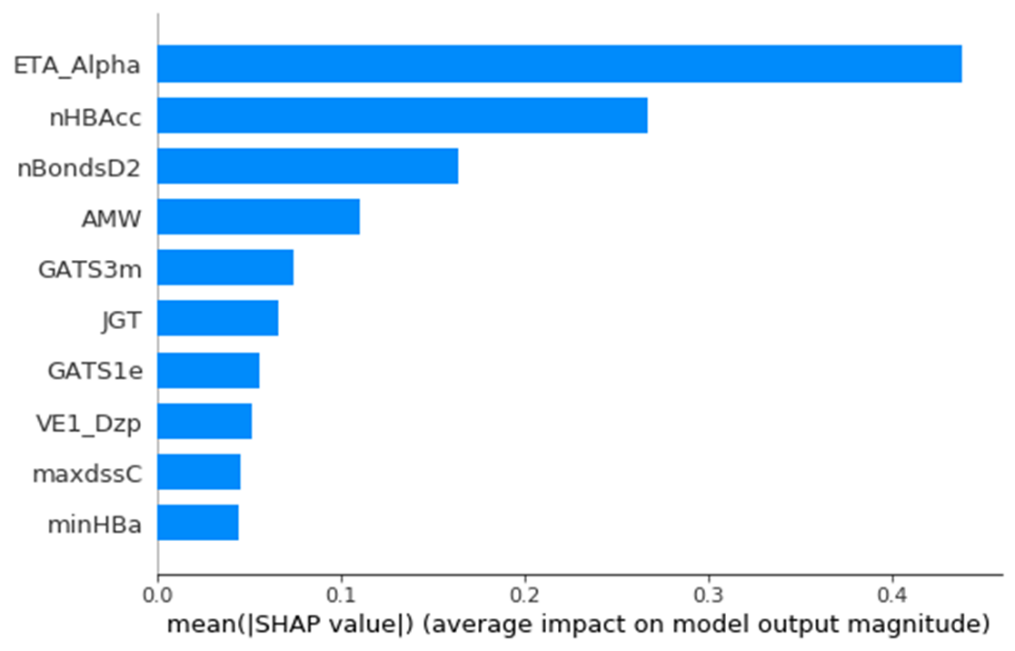


**Deep neural network**

TH_90 data subset

**
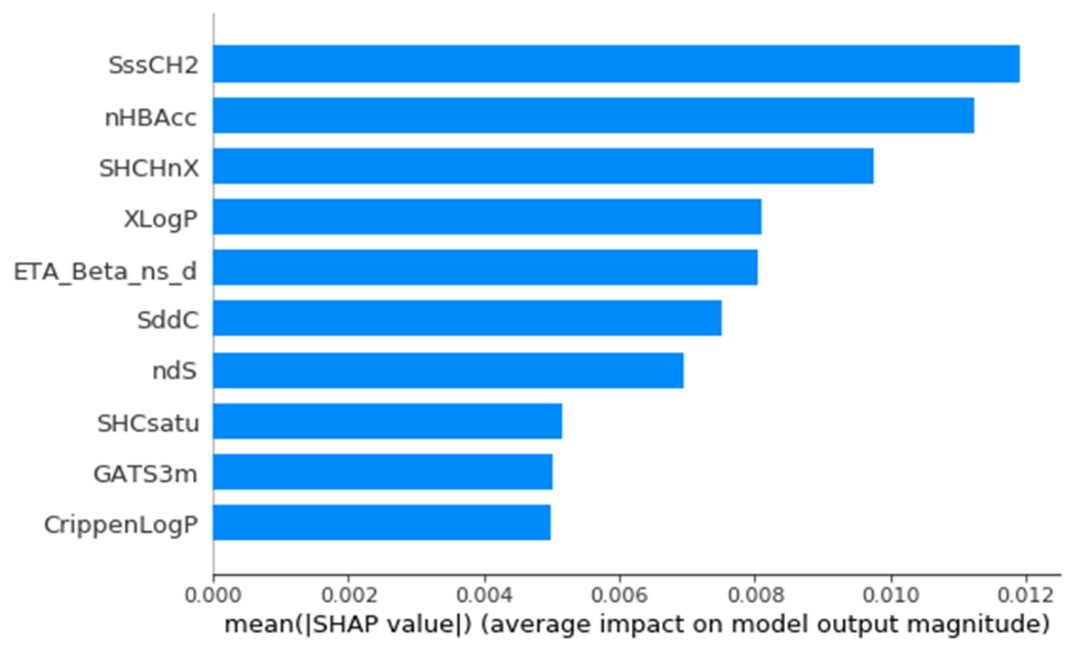
**

TH_50 data subset

**
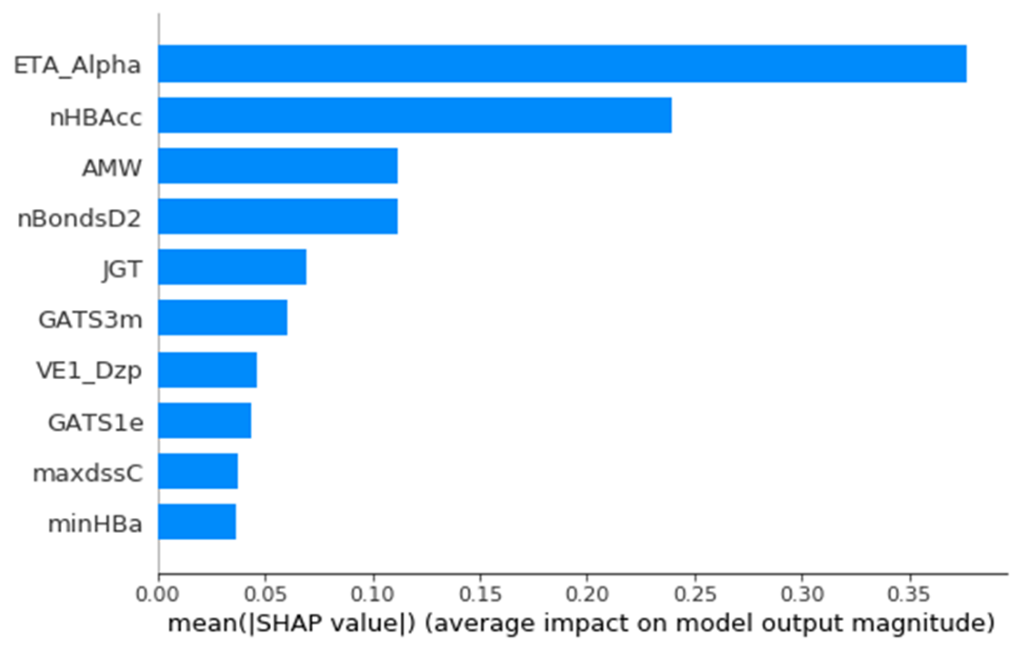
**
