# Supplementary material for: Guidance for good practice in the application of machine learning in development of toxicological quantitative structure-activity relationships (QSARs)
Source: PLoS One. 2023 May 10;18(5):e0282924. doi: 10.1371/journal.pone.0282924 (PMC10171609; doi:10.1371/journal.pone.0282924)
Supplement: Attachment — Submitted filename: Belfield et al. response to reviewers.docx [file pone.0282924.s007.docx]

We would like to thank the reviewers for their insightful comments. Taking into account the suggestions and advice offered, we have implemented extensive revision to our submission. Primary amendments include:

- Changing of title from “Good practice for machine learning methods in predictive toxicology” to “Guidance for good practice in the application of machine learning in development of toxicological quantitative structure-activity relationships (QSARs)”.
- Switching of corresponding author from Mark T.D. Cronin to James W. Firman.
- Incorporation into Section 2.1 of distinct unit dealing explicitly with dataset pre-processing.
- Inversion of Section 2.4 and 2.5 ordering.
- Expansion of this new Section 2.4 (“Statistical performance metrics and evaluation of model predictivity”) in order to present formal definition of relevant terms.
- Content formerly within Section 3.1 (“Analysis of Datasets”) relocated to form basis of new Section 3.2 (“Quality and consistency of training data”).
- Previous Section 3.2 (“Descriptor Selection”) thinned down. Much of its remaining content is presented in new Section 3.1 (“Impact of descriptor quantity upon model performance”).
- Previous Section 3.6.4 (“Extending the uncertainty assessment criteria to better evaluate ML models”) removed. Contents now dispersed across 3.6.1, 3.6.2 and 3.6.3.
- Deletion of previous Table 1, incorporation of new table (Table 4) offering more easily-readable summary of points relating to update of QSAR uncertainty scheme.
- Removal of Figure 7.
- Breaking up of Excel file within which supplementary data was previously held (this is now presented across a series of documents).
- Standardisation of terminology relating to statistical metrics, modelling approaches etc.

Detailed responses (in bold) to specific comments (bullet-points) are presented beneath.

**Reviewer #1:** I like the general tone of this paper to provide the causal interpretation of machine learning models for toxicology and demonstrating that the current level is far away from needed.

- If the goal of this paper is to present the status of machine learning (ML) methods in toxicology relative to the traditional regression analysis, then this paper satisfies it. If the goal is deeper to give a guidance what needs to be done in machine learning to reach needed causal level in toxicology, then this goal is not reached from my viewpoint. Please clarify the goal. For the second goal it can be useful to get more specific considerations of inconsistency of the mechanistic/causal interpretation with ML models to get reasons of inconsistencies like dealing with attributes, which have low scores or too many attributes for humans to see causal relations.

**The goal of the paper is now expressed in a more explicit manner within the manuscript: that is, to develop guidance for best practice when applying machine learning approaches in the development of predictive toxicology QSAR (by extension, supplementing the Cronin et al. [2018] uncertainty assessment criteria). Please refer to lines 43-48 and 113-123. Areas of particular relevance when considering factors likely to impact confidence in machine learning models are given closest attention: these being the reproducibility, interpretability and generalisability of algorithms. As such, whilst means of enhancing interpretability are discussed, we make no claim that these are capable of establishing causality in all cases. Indeed, lines** **538-540, 596-606, 689-696 and 755-757 address specifically the requirement for integration of mechanistic knowledge in order that feature importance outcomes might be rationalised (knowledge which may very often not exist). The concept (and potential necessity) of feature reduction as a route towards aiding interpretability in instances whereby identified descriptors exhibit low importance scores is introduced in lines 368-373, before appearing in 534-538 and again throughout Section 3.5.2.**

- Fig 1.
- Review. It is strange to see deep learning outside of supervised and unsupervised learning.

**Figure now revised. Distinction now drawn only between supervised and unsupervised learning forms.**

- *153. These data were reduced to 1,995 substances following the removal of duplicates.*
- *363. Over 1,000 descriptors were calculated very rapidly in this study, as is common with most ML.*
- *368. This identified 936 significant descriptors for the Tetrahymena dataset, and 1,087 descriptors for the LD50 dataset.*
- Review: Please provide in a single place the exact number of substances/cases and dimensions/descriptors of these data for each model and discuss the possible overfitting depending of the ratio between these numbers.

**Please refer to revised Table 2 (previously Table 3). This represents an expansion of what was, in the previous draft, labelled as Table 3. Number of substances present in each dataset is presented, as is the quantity of features found within each of the various subsets. Columns containing cross-validated (R^2^_CV_) and training set (R^2^_train_) R^2^ values relating to the various models are incorporated. Measure of the extent of overfitting (R^2^_over_) is provided – which is determined through the subtraction of R^2^_CV_ from R^2^_train_.**

**Influence of feature number, dataset consistency (and similar) upon observed model overfitting is discussed within 443-454.**

- *222. space between neighbours is measured by an appropriate distance metric.*
- *232. NNs are taught through an iterative process, where connection weights and biases between neurons are modified using a back-propagation algorithm that aims to minimise the error function [39].*
- *628. Certain ML methods, such as decision trees and KNN, which researchers have used exhaustively, may already be classified as interpretable, where the logical algorithm structure enable feature importance’s to be deduced [48].*
- Review. Please make these and other summaries of ML methods deeper. Finding “appropriate distance metric” is an open problem for K-NN for decades. It is very sensitive to data scaling, and some distances have no meaning for heterogeneous data like blood pressure and temperature, which are common in ML. Please avoid following simplified sources and outline advantages and disadvantages of methods not only what they compute.

**Detailed consideration of the workings behind these approaches, combined with an assessment of their merits and shortcomings, are elements which both lie beyond the scope of this study. Given the already lengthy nature of the manuscript, the addition of further technical information, readily accessible to the interested reader, shall not serve in its best interests.**

**Further general citations are added throughout Section 2.3 – whilst the issue of *k-*NN distance definition is now addressed specifically line 211. Model-specific approaches for feature importance-detection applicable to random forest and extreme gradient boosting are referenced within Section 3.5 (lines 510-512).**

- *302. SHAP is a recently developed method at the cutting-edge of model interpretability originating from the Shapley values of cooperative game theory [43]. Shapley values provide a unique method to attribute a model’s outputs towards feature contribution, and therefore guarantees the satisfaction of the three important properties: local accuracy, missingness, and consistency [44]. SHAP values are assigned for each feature for individual predictions…*
- *509. [61] advocated against traditional permutation importance methods, finding that they can give rise to misleading results particularly while dealing with correlated features. Therefore, to unearth stronger and more confident results for interpretability, an additional approach defined as Shapley Additive explanations was undertaken*
- *527. from Shapley values that originate from coalitional game theory. This method enables the pay-out (i.e., the prediction) to be fairly distributed among the players (descriptors); thus, allowing the contribution each presents to be quantified [48].*
- Review. While some ML methods are presented without their pluses and minuses, SHAP is presented with this information, but unfortunately it is misleading. It must reflect critiques of SHAP in the ML literature, see [R1-R4]. In particular, SHAP brought from the cooperative game theory in economics the assumption that the evaluation/cost/award function in ML has the same nature as a monetary award in economics that must be split between players, which is expressed formally via additivity. This is at least questionable in ML for typical heterogeneous data [R1], which can make uniqueness of SHAP irrelevant for many ML tasks. You pointed out that permutation can be misleading and should do the same with Shapley Additive explanations and analyze its results critically too. You should justify “unearth stronger and more confident results” for it or make this statement softer.

**With reference to the publications suggested, the potential shortcomings of SHAP are now addressed directly in lines 557-560. The technique is no longer presented as being necessarily superior to any other – with mention of it having capacity to “unearth stronger and more confident results” now removed.**

- *435. Folds ranged from 2 to 25 were investigated with each ML algorithm. … For all ML methods, cross validation demonstrated that the performance of all models was poorer with a low number of folds i.e., up to five.*
- *769. 10-fold validation, or thereabouts, is optimal for cross validation. Less than this and the model performance is adversely affected, more than this adds little value.*
- Review. Please provide the number of cases in each fold in experiments with different number of folds. While k-fold average is a typical way to evaluate the quality of the ML model, now the growing interest is associated with the worst-case splits to folds [R5], which is more appropriate for the task with the high cost of error, and it seems the some toxicology tasks are among those ML tasks.

**Information relating to quantity of substances-per-fold is now presented within Supplementary Material 1. Although we have not integrated the worst-case splits to folds approach into our own analysis, reference to it is incorporated within the manuscript (accompanied by the suggested citation). This may be found within lines 440-442.**

- *489. understanding of which features are providing the greatest value to each algorithm. Hence, gathering this information requires the calculation of the importance of features.*
- Review: while it is useful, it does not make the model interpretable for the end user/domain expert as you discuss in 570-576. It requires that the domain expert confirms the order of importance of features from the toxicology viewpoint, which can be spurious. Please comment more specifically is the order acceptable from the toxicology viewpoint. In essence a usability study is needed.

**Points relating to the insufficiency of the mere identification of most important features, when seeking to interpret machine learning models, are considered at several points within the updated manuscript. Particularly, across lines 538-540, 596-606, 689-696 and 755-757.**

- *628. Certain ML methods, such as decision trees and KNN, which researchers have used exhaustively, may already be classified as interpretable, where the logical algorithm structure enable feature importance’s to be deduced [48].*
- Review. It is widely accepted that decision trees and logical rules are interpretable. It is not clear for me why you do not present them in the paper analyzing only methods, which have difficulties to be interpreted. Their comparison in accuracy and interpretability with the decision trees will show if any of them have advantages. I feel that it should be done.

**Revision to text has since removed allusion to any particular modelling technique as inherently “interpretable”.**

**Regarding lone decision trees: we have excluded them owing to prior experience informing us that they are inadequate for the handling of datasets displaying the magnitude and complexity of those modelled within the study.**

**By way of illustration, we have trained decision tree models (default hyperparameters) upon the TH_90 and TH_30 data subsets. Outcomes are described within the below table:**

| **Dataset** | **R^2^_train_** | **R^2^_CV_** | **R^2^_over_** |
| --- | --- | --- | --- |
| TH_90 | 1.00 | 0.537 | 0.463 |
| TH_30 | 1.00 | 0.260 | 0.740 |

**As may be seen, values of R^2^_CV_ are far inferior with respect to those acquired through other methodologies (please refer to Table 2 within manuscript). Even the weakest-performing of the alternative models (*k*-NN in each instance) exhibit noticeably greater predictive capacity: outranking the decision trees by 0.123 (TH_90) and 0.253 (TH_30). However, it is in liability towards overfitting that the approach truly stands out – as testified by the perfect R^2^_train_ and resultant hugely inflated R^2^_over_ quantities.**

- 654-668 Review. Please make this part clearer. It is not clear what you want to say about the causal relations in the toxicology and the interpretability of the attributes. Do you want to say that of those attributes which are shown as a most informative in the algorithms give you a clue to explore a cause.

**Essentially, yes: identified features can potentially provide a first link towards unravelling the relationship between molecular characteristics and toxicological outcome. Although the initial referenced statement has been rewritten, the requirement of broader mechanistic understanding in aiding ultimate model interpretation is a point considered at several points within the revised manuscript (see once more lines 538-540, 596-606, 689-696 and 755-757).**

- 719-747 Review. As I already pointed out, you could be more cautious saying that linear regression models are fully interpretable. It is not always the case for the heterogeneous data [R1].

**Revision to text has since removed allusion to any particular modelling technique as inherently “interpretable”.**

References

- [R1] https://ddec1-0-en-ctp.trendmicro.com:443/wis/clicktime/v1/query?url=https%3a%2f%2farxiv.org%2fpdf%2f2009.10221&umid=3c9d1488-4a44-434c-91ca-134dfda3366c&auth=6b639a990a359ff1d6cc8761081d57748ce3c81e-4dd67b51bd67d1ed0e4995905798c487abd1714c
- [R2] David S. Watson, Conceptual challenges for interpretable machine learning, Synthese (2022) 200:65
- https://ddec1-0-en-ctp.trendmicro.com:443/wis/clicktime/v1/query?url=https%3a%2f%2flink.springer.com%2fcontent%2fpdf%2f10.1007%2fs11229%2d022%2d03485%2d5.pdf&umid=3c9d1488-4a44-434c-91ca-134dfda3366c&auth=6b639a990a359ff1d6cc8761081d57748ce3c81e-9c2b04329ddb62279c10c064045d5f5e3d38ee98
- [R3] Watson DS. Rational Shapley Values. arXiv preprint arXiv:2106.10191. 2021 Jun 18.
- [R4] Fryer D, Strümke I, Nguyen H. Shapley values for feature selection: The good, the bad, and the axioms. IEEE Access. 2021;9:144352-60.
- [R5] C Recaido, B Kovalerchuk, Interpretable Machine Learning for Self-Service High-Risk Decision-Making, 2022 26th International Conference Information Visualisation, Vienna, https://ddec1-0-en-ctp.trendmicro.com:443/wis/clicktime/v1/query?url=https%3a%2f%2farxiv.org%2fpdf%2f2205.04032&umid=3c9d1488-4a44-434c-91ca-134dfda3366c&auth=6b639a990a359ff1d6cc8761081d57748ce3c81e-3f855f08452ad66423bc9830de3c217813bdc14f

**Each publication now cited within manuscript.**

**Reviewer #2:** The study main aim is to provide a well formed framework to be considered as a best practice methodology for toxicology prediction. Different models were established with a variety of assessment methods to assure correct and efficient results. Model building also included the steps to construct feature importance which would be of great importance for easier and more efficient prediction of chemical compounds toxicity. The development of such models would guide the studies in the same domain with a clear workflow that was shown practically through this study.

I suggest considering the manuscript for publication but I greatly recommend considering the comments. Mainly, reforming many sentences is important to get the ideas easier.

- In addition, a more focused description of the ideas and direct link to the experimental results would be helpful to make the reader understand and get the benefit from the research.

**Following rewrite, we believe that such associations are now drawn with greater clarity. Section 3.1 more clearly relates consideration of feature size and performance to trends observed within our own models – whereas Section 3.2 does likewise with regards to dataset quality and consistency. Section 3.3 considers overfitting in terms of their R^2^_over_ variation, Section 3.4 invokes impact upon predictivity of each as sustained following hyperparameter optimisation, and Section 3.5 provides a practical demonstration of feature reduction and its potential in enhancing general interpretability.**

- General English enhancements and corrections are recommended for more clear explanations of ideas. Generally, long sentences are frequent through the manuscript making it difficult to get the ideas.

- Please consider rewriting or clarifying the following sentences:

**All identified statements have been rewritten over course of manuscript update.**

- The following comments are recommended to be considered:

- I would recommend adding more recent work about using ML in toxicology prediction from the literature.

**Reference to the 2022 review of Lin and Chou, covering all recent developments in this field, is now provided within Introduction (**[**https://doi.org/10.1093/toxsci/kfac075**](https://doi.org/10.1093/toxsci/kfac075)**).**

- Additional details about dataset preprocessing would be useful

**Section 2.1 has since been restructured. Such details are now provided (lines 131-137).**

- *170: “Redundant descriptors were removed that were either uninformative or unstable.”*
- Redundancy could be understood as repetitions, but on what basis you decided uninformative or unstable descriptors?

**Revised text now reads: “Uninformative or unsuitable (and hence redundant) descriptors were removed – including those containing missing values, or else exhibiting low variance”.**

**In essence, these terms relate to descriptors that are either constant or near-constant in value, or are else outright unusable owing to their incompatibility with the modelling approach. The former tended to be uniformly zero (such as “number of neodymium atoms”), whereas the latter would generally incorporate missing quantities. “Unstable” would have been a typo.**

- *176: “Pairwise correlation coefficients values used to limit collinearity and create the subsets were: 0.9, 0.8, 0.7, 0.6, 0.5, 0.4, and 0.3. The descriptor of the pair that reported the weakest correlation to the target was omitted.”*
- This is not completely clear, on what basis did you decide the correlation thresholds to drop descriptors correlated to the target?

**We chose these values on account of them being round numbers – which in addition served to grant effective spreads in terms of the quantity of features remaining across each of the various subsets.**

- Also, you assumed complete independence between descriptors by doing this, which is not always correct.

**Assessment of collinearity provided a means through which the extent of (in)dependence may be ascertained.**

- Please add the proper citation for the sci-kit learn library when mentioned.

**Details now provided upon first mention of resource (lines 158-159).**

- 218: Add reference.

**Section 2.3.3 has since been re-written. Two citations are provided, covering the details presented.**

- Many details in the results section is better to be moved to the data and methods section:

**Following extensive rewriting of both sections, much of this detail has accordingly been transferred.**

- 3.1: Analysis of Datasets

**Details relating to the quality of datasets have been retained within the Results and Discussion section (see repurposed 3.2, titled “Quality and consistency of training data”). They are now linked explicitly to patterns in predictivity (throughout Section 3.2), and to overfitting potential (lines 433-454) amongst the models trained.**

- 3.5: Many details in the subsections under the feature importance section.

**Excess detail has been removed, particularly as relates to SHAP. Description of principles now confined, in both instances, to Section 2.6.**

- 3.6.1 Reproducibility: being a subsection within the results and discussion section, what are the results mentioned or discussed in this section, please make it clear, if any.

**No experimental treatment of reproducibility was afforded through this study. As such, the forwarding of guidance (in the form of updates offered to appropriate uncertainty assessment criteria) was informed solely through theoretical extrapolation of factors know as essential in ensuring the replicability of standard QSAR. The contents of Section 3.6.1 reflect this – discussing those machine learning-specific considerations relevant to the topic.**

- In all subsections 3.6.1,3.6.2, 3.6.3, I would suggest reducing the theoretical description of each criteria to the least possible. I Also suggest that most of the derails in these sub-sections can be moved to the methods section while removing any redundancy.

**Within Sections 3.6.2 and 3.6.3, explicit definition of the concepts under consideration (interpretability and generalisability) has now been stripped to a bare minimum. References to their earlier discussion within the manuscript are instead now provided. Since no equivalent experimental treatment of model reproducibility is given, its mention prior to this point is less substantial. As such, it is necessary to present a slightly more robust treatment of its basic principles as part of 3.6.1.**

- The subsections did not mention the results that prove how the suggested models satisfies these assessment criteria.

**The models presented within this study were trained for the purposes of illustrating means through which a machine learning QSAR (as applied to toxicology) might be made, in a relative sense, more or less interpretable or generalisable. Essentially, they are simply tools supporting the primary intent of the work: this being the development of a guidance framework. In absolute terms, none is intended to serve as an embodiment of an ideal model.**

- 370: What was the basis on which you selected these collinearity levels?

**We chose these values on account of them being round numbers – which in addition served to grant effective spreads in terms of the quantity of features remaining across each of the various subsets.**

- Please indicate clearly the subsets of data used for training and testing the ML models. Did you keep an independence test set? Or you only used cross-validation?

**Cross-validation was used to evaluate model performance upon unseen data. Please see Sections 2.4 and 3.3, alongside Supplementary Table 1, for further detail.**

- 463: Could you highlight what were the hyperparameter values that resulted in the best performing models?

**Additional information relevant to optimisation of model performance – including those optimum hyperparameter values determined – are presented within expanded Table 1 (was previously Table 2). For further detail, please refer to Supplementary Materials 2, 3 and 4.**

- In Table 4, please highlight the best results to be easier to find.

**Colour-coding is now introduced across Tables 2 and 3 (previously Tables 3 and 4), indicative either of model performance or the influence of optimisation.**

- Please show how optimization improved the prediction results by pointing out the difference between the model performance before and after optimization.

**Table 3 (previously Table 4) now explicitly outlines variations in performance relative to default.**

- What is your comment on the wide gap between training and testing R2 in most of the approaches, how was this gap affected by optimization.

**This property, since labelled R^2^_over_, is now discussed explicitly at various points within the manuscript (in which it serves as indicator of the extent to which a model is overfit). Please refer to Sections 2.4, 3.3 and 3.6.3 by way of example. In relation specifically to optimisation, Section 3.4 (particularly lines 489-498) and Table 3 should be referred to.**

- 580: “The uncertainty criteria were grouped into ten components that summarise the main characteristics of a QSAR …” please add few details to make the context clear.

**Statement now rewritten (these ten components are no longer given mention). Please refer to lines 611-619 for provision of background relating to the uncertainty assessment scheme.**

- Please provide a better resolution for figures from Figure 2 to Figure 6.

**Updated figures should now display higher resolution.**

- Table 10 is difficult to follow in the current format, please consider a different way of organization for better display of information.

**Table 4 (previously Table 10) now presented in truncated form within manuscript. Unabridged version relocated as Supplementary Material 6.**

- Please highlight in more details the most important descriptors that were by most of the techniques and define their chemical effect briefly when possible.

**For discussion of specific identified descriptors, and how these might vary in accordance with either the machine learning or the feature importance approach adopted, please refer to lines 524-534, 553-555, 560-570, 587-603.**

**Reviewer #3:** Authors presented a well written thesis on the best practice of machine learning for toxicity prediction.

- Introduction, pp 7-8, lines 103-120: This paragraph currently seemed to be floating out of context, it is recommended to move the paragraph to Line 77 after the first paragraph.

**Introduction is now restructured. As such, this paragraph no longer exists in a recognisable form.**

- 3. Methods, 2.1 Data curation: It is advised that the author should publicly share both the original dataset and the curated dataset used in the study along with the Python code used for ML model building as to abide by the PLOS journal guidelines (see Data Availability https://ddec1-0-en-ctp.trendmicro.com:443/wis/clicktime/v1/query?url=https%3a%2f%2fjournals.plos.org%2fplosone%2fs%2fdata%2davailability&umid=3c9d1488-4a44-434c-91ca-134dfda3366c&auth=6b639a990a359ff1d6cc8761081d57748ce3c81e-03a9d9320596746891e4e729bb8da103f745bfd9 and Sharing Code https://ddec1-0-en-ctp.trendmicro.com:443/wis/clicktime/v1/query?url=https%3a%2f%2fjournals.plos.org%2fplosone%2fs%2fmaterials%2dsoftware%2dand%2dcode%2dsharing&umid=3c9d1488-4a44-434c-91ca-134dfda3366c&auth=6b639a990a359ff1d6cc8761081d57748ce3c81e-078375ceac3f738fb31fd5c121b22ebf084267a4) in order to facilitate research reproducibility as well as benefitting the scientific community. The author did a good job of highlighting this importance in section 3.6.1 Reproducibility and it would be great if this manuscript could publicly share the data and code. In regards to the dataset, this would possibly include the compound SMILES, its corresponding bioactivity value and reference source.

**Link to GitHub repository containing both the adopted source code and the modelled datasets is now provided within manuscript (line 129)**.

- The ML models reported in the manuscript was well carried out and explained in a structured and logical manner.

- The presented guideline is extremely helpful for practitioners in future QSAR model building.

- It is recommended that the authors cite a related paper for the point on research reproducibility <https://ddec1-0-en-ctp.trendmicro.com:443/wis/clicktime/v1/query?url=https%3a%2f%2fjcheminf.biomedcentral.com%2farticles%2f10.1186%2fs13321%2d020%2d0408%2dx&umid=3c9d1488-4a44-434c-91ca-134dfda3366c&auth=6b639a990a359ff1d6cc8761081d57748ce3c81e-64f623e87b1d93656220db72f52c429332ca6918>

**Citation now added.**

- 3. Results and Discussion, 3.6.2 Interpretability, Line 645: Should the term "casually" in the phrase "casually related towards the outputs" a typo and should it be "causally"?

**Although general restructuring has occurred within this section, the typo is nevertheless now amended.**

- - 3. Results and Discussion, 3.6.2 Interpretability: Could the authors provide recommendations on how readers could go about interpreting the important descriptors shown in Figures 4-6.

**The application of mechanistic knowledge (where present) in contextualising outcomes of feature importance analysis is discussed at several points within the revised manuscript, including lines 596-606, 689-698, 755-757.**
